# Interventions to ameliorate reductions in muscle quantity and function in hospitalised older adults: a systematic review towards acute sarcopenia treatment

**DOI:** 10.1093/ageing/afaa209

**Published:** 2020-10-24

**Authors:** Carly Welch, Zeinab Majid, Carolyn Greig, John Gladman, Tahir Masud, Thomas Jackson

**Affiliations:** Institute of Inflammation and Ageing, University of Birmingham, Birmingham B15 2TT, UK; Medical Research Council and Versus Arthritis Centre for Musculoskeletal Ageing Research, University of Birmingham and University of Nottingham, Nottingham, UK; University Hospitals Birmingham NHS Trust, Birmingham, UK; Institute of Inflammation and Ageing, University of Birmingham, Birmingham B15 2TT, UK; University Hospitals Birmingham NHS Trust, Birmingham, UK; Medical Research Council and Versus Arthritis Centre for Musculoskeletal Ageing Research, University of Birmingham and University of Nottingham, Nottingham, UK; University Hospitals Birmingham NHS Trust, Birmingham, UK; School of Sport, Exercise, and Rehabilitation Sciences, University of Birmingham, Birmingham B15 2TT, UK; Medical Research Council and Versus Arthritis Centre for Musculoskeletal Ageing Research, University of Birmingham and University of Nottingham, Nottingham, UK; National Institute for Health Research Birmingham Biomedical Research Centre, University Hospitals Birmingham NHS Foundation Trust and University of Birmingham, Birmingham, UK; Medical Research Council and Versus Arthritis Centre for Musculoskeletal Ageing Research, University of Birmingham and University of Nottingham, Nottingham, UK; National Institute for Health Research Nottingham Biomedical Research Centre: Musculoskeletal Disease theme, Nottingham, UK; Healthcare of Older People, Queens Medical Centre, Nottingham University Hospitals NHS Trust, Nottingham, UK; Institute of Inflammation and Ageing, University of Birmingham, Birmingham B15 2TT, UK; Medical Research Council and Versus Arthritis Centre for Musculoskeletal Ageing Research, University of Birmingham and University of Nottingham, Nottingham, UK; University Hospitals Birmingham NHS Trust, Birmingham, UK

**Keywords:** acute sarcopenia, systematic review, older people, interventions

## Abstract

**Objective:**

Assimilate evidence for interventions to ameliorate negative changes in physical performance, muscle strength and muscle quantity in hospitalised older adults.

**Methods:**

We searched for articles using MEDLINE, Embase, CINAHL and Cochrane library using terms for randomised controlled trials, older adults, hospitalisation and change in muscle quantity, strength or physical performance. Two independent reviewers extracted data and assessed risk of bias. We calculated standardised mean differences for changes in muscle function/quantity pre- and post-intervention.

**Results:**

We identified 9,805 articles; 9,614 were excluded on title/abstract; 147 full texts were excluded. We included 44 studies including 4,522 participants; mean age 79.1. Twenty-seven studies (*n* = 3,417) involved physical activity interventions; a variety were trialled. Eleven studies involved nutritional interventions (*n* = 676). One trial involved testosterone (*n* = 39), two involved Growth Hormone (*n* = 53), one involved nandrolone (*n* = 29), and another involved erythropoietin (*n* = 141). Three studies (*n* = 206) tested Neuromuscular Electrical Stimulation. Evidence for effectiveness/efficacy was limited. Strongest evidence was for multi-component physical activity interventions. However, all studies exhibited at least some concerns for overall risk of bias, and considering inconsistencies of effect sizes across studies, certainty around true effect sizes is limited.

**Conclusion:**

There is currently insufficient evidence for effective interventions to ameliorate changes in muscle function/quantity in hospitalised older adults. Multiple interventions have been safely trialled in heterogeneous populations across different settings. Treatment may need to be stratified to individual need. Larger scale studies testing combinations of interventions are warranted. Research aimed at understanding pathophysiology of acute sarcopenia will enable careful risk stratification and targeted interventions.

## Key points

- A variety of interventions have been trialled for outcome measures relevant to acute sarcopenia.
- There is currently insufficient evidence for effective interventions to treat acute sarcopenia.
- Trials involving a combination of interventions in stratified individually are warranted.

## Introduction

Sarcopenia is defined by low muscle strength with low muscle quantity/quality; additionally demonstrated low physical performance defines severe sarcopenia. Cut-offs are two standard deviations (SDs) below means of young healthy reference populations [1]. Acute sarcopenia (acute muscle insufficiency) particularly affects hospitalised older adults [2,3]. Normally proceeded by stressor events, it is defined by acute declines in muscle quantity/quality and/or function (strength or physical performance) producing incident sarcopenia [1,3]. Previous reviews considered chronic sarcopenia treatment/prevention [4–6]; strongest evidence exists for physical activity. Resistance training improves muscle quantity, strength and physical performance in community-dwelling populations [7]. Some trials demonstrated enhanced benefit of nutritional supplementation alongside [8]. Large studies are underway evaluating combined nutritional and exercise interventions for chronic sarcopenia [9].

It is unknown whether chronic sarcopenia interventions can treat acute sarcopenia. Mechanisms differ, which may affect treatment efficacy. Acute sarcopenia is associated with greater systemic inflammation and immune-endocrine dysregulation. Inflammation (acute or chronic) may blunt response to exercise or protein challenges (anabolic resistance), but this may be acutely/severely upregulated in acute sarcopenia [10]. Acute sarcopenia follows an accelerated course [3]; traditional treatments may not work fast enough. Additionally, community interventions may be unfeasible in hospital. This review aimed to identify trialled interventions for ameliorating negative changes in muscle quantity, strength or physical performance in hospitalised older adults, and to summarise/synthesise findings.

## Methods

### Protocol and registration

Protocol was agreed by all researchers and registered with Prospective Register of Systematic Reviews—CRD42018112021. Reporting is consistent with Preferred Reporting Items for Systematic Reviews and Meta-Analyses guidance.

### Eligibility criteria

We included randomised controlled trials (RCTs) and quasi-RCTs involving hospitalised patients ≥65 years-old, where pre- and post-intervention measurements of muscle quantity, strength or physical performance were available. Post-intervention measures until 28 days post-intervention were included. We included physical activity, nutritional, pharmaceutical or Neuromuscular Electrical Stimulation (NMES) trials. Exclusion criteria were: degenerative neuromuscular disorders; acute stroke; trials of parenteral nutrition, surgical technique/invasive procedure, chemotherapy/radiotherapy, or anaesthetic agents/techniques; no control group; lengths of stay less than 2 days. We included studies that measured muscle quantity using computed tomography (CT), magnetic resonance imaging (MRI), dual energy X-ray absorptiometry (DXA), bioelectrical impedance analysis (BIA), or ultrasound, muscle strength using handgrip strength, knee flexion, or knee extension, or physical performance using short physical performance battery (SPPB), gait speed, timed up and go (TUG), or 6-Minute Walking Test (6MWT). There were no date or language restrictions.

### Information sources

We searched electronic databases (MEDLINE, Embase, CINAHL, CENTRAL) on 16 January 2019; search repeated on 3 April 2020. Grey literature was identified through Web of Science, Google Scholar, Clinicaltrials.gov, article references and protocol citations. We contacted authors for information where necessary, including requesting age breakdown of data. If no response was obtained, a decision was made to include studies where mean age was one SD > 65.

### Search strategy

We used published and unpublished terms for study design (RCTs), population (older adults AND hospitalised) and outcome measures (muscle mass OR muscle strength OR physical performance) in our search. Full search strategy is available in the online supplement; this was reviewed and agreed with an information specialist.

### Study selection

Citations were imported into Microsoft Excel 2016. Duplicates were removed automatically/manually. Two reviewers independently screened titles and abstracts for inclusion (CW, ZM). Disagreements were resolved through discussion. Full texts were reviewed independently by the same reviewers; disagreements were resolved through discussion or third review (TAJ).

### Data extraction

Data were extracted independently by two reviewers (CW, ZM) using a template (Microsoft Excel 2016). Extracted data were country, study design, sample size and dropouts, sample characteristics (age, ethnicity, body mass index—BMI, sex), speciality, intervention description (type of intervention, how delivered), intervention characteristics (timing of intervention, dosage), control group, outcome data, length of stay and adverse events. Outcome data at baseline and follow-up to include muscle quantity, muscle strength and physical performance were extracted.

### Risk of bias

Two reviewers (CW, ZM) independently assessed risk of bias using Cochrane risk of bias tool. Conflicts were resolved by discussion. Risk of bias was collated using RevMan version 5.3 [11].

### Synthesis of results

We summarised study and participant characteristics, and outcome data at baseline and follow-up using means/SDs in text and tables. Interventions were grouped by subtype and outcomes. All studies were included in narrative synthesis. If sufficient information was available to estimate standardised mean differences (SMDs) of change scores, effect sizes were evaluated as described in statistical analysis section. Certainty of interventions with large effect sizes was evaluated using Grading of Recommendations, Assessment, Development and Evaluations [12].

### Statistical analysis

Correlations for outcome measures were calculated from studies reporting SDs of change scores and baseline/follow-up measures [13]. Mean correlation for each outcome was used to estimate SD of change in outcomes in studies where this was not available. We calculated SMDs of change scores by dividing difference in change score between comparison and intervention groups by SD of change score in comparison group [14]. Effect sizes were calculated to one decimal place and classified as no effect (≤0.1), small (0.2–0.4), medium (0.5–0.7) or large (0.8 or greater) [15]. If more than one effect size was available for a single trialled intervention and outcome type, the larger was included. Meta-analysis was not performed due to high heterogeneity of interventions and outcomes.

**Figure 1:**
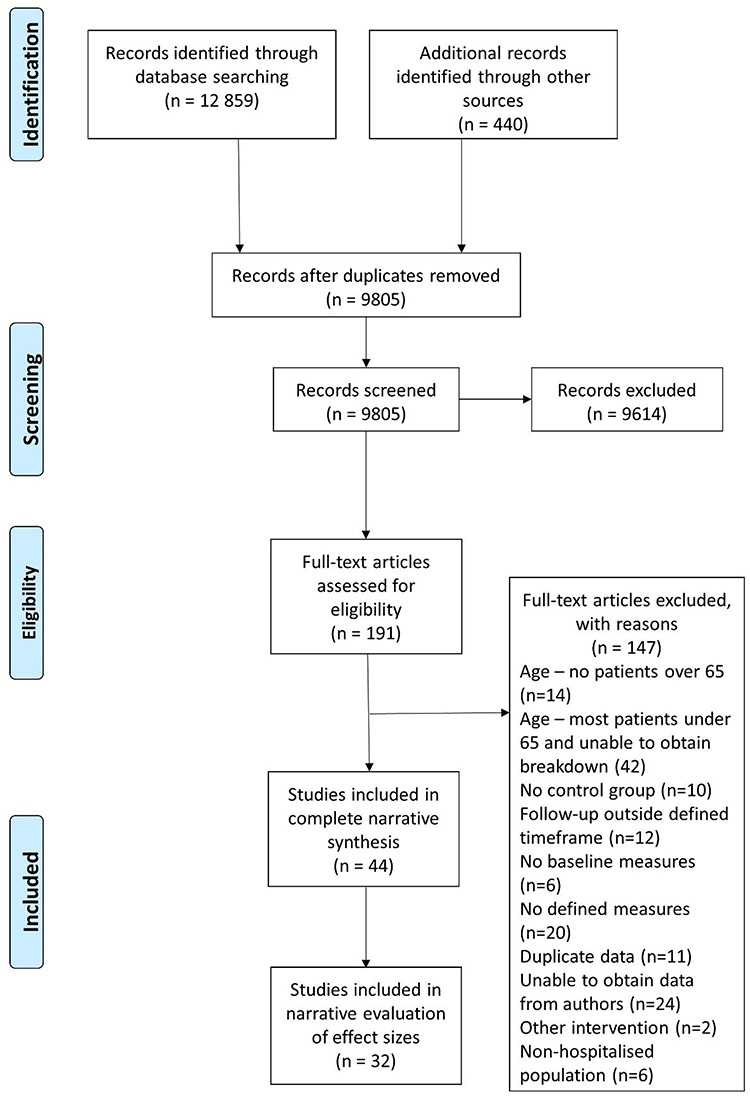
Flowchart demonstrating identification of included studies. All stages of screening and inclusion/exclusion were performed in duplicate. Reasons for exclusion of articles reviewed as full texts are specified.

## Results

### Study selection

We identified 9,805 articles after duplicates removal. We excluded 9,613 following title/abstract screening; 192 full texts assessed for eligibility. We excluded 148 full text articles due to mean age not more than one SD above 65 (*n* = 56), no control group (*n* = 10), follow-up over 28 days (*n* = 12), no baseline measures (*n* = 6), no measures meeting inclusion criteria (*n* = 20), duplicate data (*n* = 11), unable to obtain necessary data from authors (*n* = 24), other intervention type (*n* = 2), and non-hospitalised population (*n* = 6) (Figure 1). We included 44 studies in narrative synthesis and 32 studies in effect size evaluation.

### Study characteristics

This review included 4,522 participants (2,160 control, 2,362 intervention). Sample size per arm ranged from 7 to 232. Most studies were small; 52% (23/44) [17–40] included 30 or fewer participants per arm; only 9% (4/44) [41–43] included over 100 participants in both arms. Mean age across all studies was 79.1 years; 59% female. Of studies reporting BMI, 74% (20/27) [17, 18, 21, 23, 26–28, 31, 34, 35, 38, 43–51] reported mean overweight (≥25) BMI; three studies reported mean obese (≥30) BMI in at least one arm [23, 39, 52]. One study reported data on ethnicity [38]. Two studies [45, 50] reported frailty prevalence in control and intervention arms by recognised definitions; a third reported mean frailty indices [37]. Table 1 shows included studies’ details; full study characteristics and results are available online. Table 2 shows effect sizes separated by interventions and outcomes.

### Physical activity interventions

Most studies (61%, 27/44) reported physical activity interventions. Eighty-nine percent (24/27) included physical performance [17–22, 34, 37, 38, 40–47, 50, 53–58] and 44% (12/27) included muscle strength [18, 23–25, 36, 43, 44, 46, 47, 54, 57, 58]. One study reported muscle quantity change [38], a multi-arm trial including nutritional and pharmaceutical interventions. Trials were conducted in various settings including elective orthopaedic [18, 47], colorectal [20, 25, 46], orthopaedic rehabilitation [19, 21, 54, 58], vascular [17], and cardiac surgery [40, 44, 55], and geriatric [22, 24, 34, 36, 37, 42, 43, 56, 57], respiratory [23] and general medicine [38, 41, 45, 53].

A range of physical activity interventions were trialled; evidence for effect was limited. Interventions included strength and balance training [21, 38, 40, 44, 46, 56], early and/or increased mobilisation [17, 20, 22, 24, 25, 41], group exercise [42], water exercise/physiotherapy [18], chair-based exercise [34, 38], seated side-tapping [47], pedal exercisers [23, 36] and progressive weight-bearing exercise in orthopaedic rehabilitation [19, 54, 58], using specialised harnesses where appropriate. An individualised multimodal physical training programme involving resistance exercise using machines and/or weights and gait/balance training substantially improved physical performance (gait speed and SPPB) and muscle strength in one of the largest studies [43]. Other trials of individualised physical training programmes (strength with or without aerobic exercise stratified by frailty/functional status) showed small effects on physical performance [37, 45, 53, 55, 57]. Differences may relate to how interventions were delivered or adherence. The trial with the largest effect size reported adherence rates of 83.4–95.8% (≥90% exercises successfully performed each session) [43] compared to 59.7% (>3 sessions attended per week; offered daily) in another [57].

**Table 1:** Characteristics of all studies included in narrative synthesis

Author, date	Setting	*N* (control/intervention)	Intervention	Outcomes
Physical activity				
Busch [44], 2012	Cardiac surgery	64/57	Resistance and balance training	TUG 6MWT Knee extension
Blanc-Bisson^b^ [24], 2008	Geriatric medicine	24/22	Early physiotherapy	Handgrip
Braun [37], 2019	Geriatric medicine	18/17	Augmented Prescribed Exercise Programme	Gait speed TUG 6MWT
de Morton [41], 2007	General medicine	126/110	Physiotherapy-designed exercises	TUG
Deer [38], 2019	General medicine	20/21	Chair-based and resistance exercise	SPPB DXA FFM
Fiore^b^ [20], 2017	Elective colorectal surgery	22/25	Early mobilisation	6MWT
Giangregorio [19], 2009	Orthopaedic rehabilitation	7/14	Body weight supported treadmill training	TUG
Henriksen^b^ [25)], 2002	Elective colorectal surgery	12/13	Enhanced recovery	Handgrip Knee extension
Houborg [46], 2006	Elective colorectal surgery	59/60	Strength training programme	Gait speed Handgrip strength Knee extension
Jones [53], 2006	General medicine	80/80	Individualised progressive exercise	TUG
Martinez-Velilla [43], 2019	Geriatric medicine	185/185	Multi-component physical exercise	Gait speed SPPB Handgrip
McCullagh [45], 2017	General medicine	95/95	Augmented prescribed exercise programme	Gait speed SPPB Handgrip
McGowan^b^ [36], 2018	Acute medicine for older people	25/25	Pedal exerciser	Knee extension Knee flexion
Moseley [54], 2009	Orthopaedic rehabilitation	80/80	Weight-bearing exercise	Gait speed Knee extension
Ortiz-Alonso [50], 2019	Geriatric medicine	131/150	Chair-based exercise and walking	SPPB
Opasich [55], 2010	Cardiac surgery	80/160	Individualised physical training programme	TUG 6MWT
Prasciene [40], 2019	Cardiac surgery	15/14	Balance and resistance training	SPPB 6MWT
Rahmann [18], 2009	Elective orthopaedic	20/24	Aquatic physiotherapy	TUG Knee extension
		24/21	Water exercise	TUG Knee extension
Raymond [42], 2017	Geriatric medicine	232/236	High-intensity group exercises	TUG
Said^b^ [22], 2012	Geriatric rehabilitation	24/22	Enhanced physical activity	TUG
Said^b^ [56], 2018	Geriatric rehabilitation	93/98	Multimodal exercise programme	Gait speed TUG
Sano [47], 2018	Elective orthopaedic	41/40	Seated side-tapping training	Gait speed TUG Knee extension Knee flexion
Schwenk [57], 2014	Geriatric rehabilitation	74/74	Individualised physical training programme	Gait speed Handgrip
Sherrington [58], 2003	Orthopaedic rehabilitation	39/41	Weight-bearing exercise	Gait speed Knee extension
Tal-Akabi^b^ [21], 2007	Orthopaedic rehabilitation	29/33	High-intensity exercise	TUG
Torres-Sánchez [23], 2017	Respiratory	29/29	Pedal exerciser	Knee extension
Wnuk [17], 2016	Vascular	16/15	Backward walking	6MWT
		16/16	Forward walking	6MWT
Nutrition				
Beelen [48], 2017	General medicine	39/36	Protein-enriched familiar foods	SPPB Handgrip Knee extension
Bouillanne [30], 2018	Geriatric rehabilitation	14/13	Citrulline amino acid	DXA ASMM
Deer [38], 2019	General medicine	20/20	Whey protein	SPPB DXA FFM
		20/20	Whey protein and exercise	SPPB DXA FFM
Ekinci [59], 2016	Orthopaedic surgery	37/38	Beta-hydroxy-beta-methylbutyrate	Handgrip
Files [39], 2020	Critical care	11/11	Nitrate-rich beetroot juice	SPPB
Gade [49], 2019	General medicine	82/83	Protein-enriched milk supplement	Gait speed Handgrip BIA FFM
Hermanky [27], 2017	Orthopaedic surgery	20/20	Nutritional consultation and exercise	Handgrip BIA FFM
Niccoli [26], 2017	Geriatric medicine	26/26	Whey protein	Gait speed TUG Handgrip Knee extension
Ogasawara [29], 2018	Respiratory medicine	21/21	EPA-enriched oral nutritional supplements	BIA SMI
Pedersen [51], 2019	General medicine	42/43	Protein and exercise	Gait speed Handgrip
Saudny-Unterberger [28], 1997	Respiratory medicine	16/17	Oral nutritional supplements	Handgrip
Pharmaceutical				
Deer [38], 2019	General medicine	20/19	Testosterone	SPPB DXA FFM
Hedström [32], 2004	Orthopaedic surgery	9/11	Growth hormone	Knee extension DXA LBM
Sloan [33], 1992	Orthopaedic surgery	14/15	Nandrolone	BIA FFM
Weissberger [31], 2003	Orthopaedic surgery	16/17	Growth hormone	Knee flexion CT thigh CSA
Zhang [60], 2019	Orthopaedic surgery	33/44	EPO injections (females)	DXA ASM
		25/39	EPO injection (males)	DXA ASM
Neuromuscular electrical stimulation				
Lopez-Lopez [52], 2019	General medicine	47/48	NMES and exercise combined	SPPB
Martin-Salvador [35], 2016	Respiratory medicine	20/24	Exercise and NMES combined	Knee extension
Zinglersen [34], 2018	Geriatric medicine	48/20	Chair-based functional exercise	Gait speed
		8/12	NMES and functional training	Gait speed

N, participant numbers; ASMM, appendicular skeletal muscle mass; FFM, fat-free mass; SMI, skeletal muscle index; LBM, lean body mass; CSA, cross-sectional area; LoS, length of hospital stay; COPD, chronic obstructive pulmonary disease.

^b^Unpublished data.

**Table 2:** Summary of intervention effect by intervention type, outcome type and effect size

		Physical performance				Muscle strength			
		Effect size^a^	*N* (con/exp)	Risk of Bias^b^	Study	Effect size^a^	*N* (con/exp)	Risk of Bias^b^	Study
Physical activity	Strength and balance training	+ ++ ++ +++	93/98 64/57 20/21 15/14	+ +/−—-	[56] [44] [38] [40]	−	64/57	+/−	[44]
	Early/increased mobilisation, or additional physiotherapy	-—++ ++ -	24/22 22/25 16/16 126/110 131/150	- + +/−—-	[22] [20] [17] [41] [50]	- +++	24/22 12/13	- -	[24] [25]
	Water exercise and physiotherapy	+	20/24	+/−	[18]	+	20/24	+/−	[18]
	Seated side-tapping	+++	41/40	−	[47]	−	41/40	−	[47]
	Seated pedal exercises	No data				+ ++	25/25 29/29	- +/−	[36] [23]
	Progressive weight-bearing exercise	+ + +++	39/41 80/80 7/14	+/− +/− -	[58] [54] [19]	- -	39/41 80/80	+/− +/−	[58] [54]
	Individualised physical training programme	- + + +++ +++	95/95 74/74 80/160 185/185 18/17	+/− +/−—+/− +/−	[45] [57] [55] [43] [37]	-—+++	95/95 74/74 185/185	+/− +/− +/−	[45] [57] [43]
Nutrition	Protein-enriched foods	+++ ++	26/26 20/20	+/− -	[26] [38]	+ +++	39/36 26/26	+/− +/−	[48] [26]
	Protein and exercise	+++	20/20	−	[38]	+	42/43	−	[51]
	β-Hydroxy-β-MethylButyrate	No data				−	37/38	+/−	[59]
	Oral nutritional supplementation and snacks					+++	16/17	+/−	[28]
	Nutrition consultation combined with exercise					+	20/20	+/−	[27]
Drugs	Testosterone	+++	20/19	−	[36]	[38] No data			
	Growth hormone	No data				−	9/11	−	[32]
NMES	NMES in combination with exercise	+++	47/48	+/−	[52]	+	20/24	+/−	[35]

*N*, participant numbers; con, comparison group; exp, intervention group.

^a^Effect sizes categorised as: no effect [−] (≤0.1), small [+] (0.2–0.4), medium [++] (0.5–0.7), or large [+++] (0.8 or greater).

^b^Risk of Bias categorised according to overall risk as: low [+], some concerns [+/−], or high [−].

Interventions that ameliorated reductions in physical performance in trial populations included backward walking [17], progressive exercises stratified by frailty [55], resistance and balance training [44], chair-based resistance exercise [38], individually progressed lower limb and core strengthening exercise [45], individualised progressive resistance, balance, and walking exercises [43] and seated side-tapping [47]. Interventions that ameliorated reductions in muscle strength included pedal exercise [23], individualised progressive resistance, balance and walking exercises [43] and early mobilisation with enhanced recovery after surgery [25]. A high-intensity physiotherapy-led group exercise programme was as efficacious as individual sessions; group exercise resulted in improved therapist efficiency [42]. Group exercise was embedded into a multimodal physical training trial [43].

### Nutritional interventions

Eleven nutrition trials were identified. Populations included orthopaedic surgery [27, 59], geriatric [26, 30], general [38, 48, 49, 51], and respiratory medicine [28, 29] and critical care [39]. Six studies reported physical performance change [26, 38, 39, 48, 49, 51], seven muscle strength change [26–28, 48, 49, 51, 59] and four muscle quantity change [27, 29, 30, 38]. Most studies were small; only one included more than 45 patients per arm. Interventions included protein-enriched foods [26, 48] or supplements [38, 49, 51], β-Hydroxy-β-MethylButyrate [59], oral nutritional supplementation [28], eicosapentaenoic acid [29], citrulline [30], nitrate-rich beetroot juice [49] and nutritional consultation [27]. Three trials combined nutritional consultation to reach specified caloric/protein intake) with strength/resistance training [27, 38, 51]. One study of progressive strength training followed by immediate protein supplementation showed statistically significant improved handgrip strength [51]. Statistically significant improvements in physical performance were demonstrated comparing all interventions in a multi-arm study to placebo, including whey protein with/without exercise [38].

### Pharmaceutical interventions

Five trials involved pharmaceuticals; four in orthopaedic surgery populations. Pharmaceuticals included growth hormone (GH) [31, 32], steroid (nandrolone) [33], testosterone [38] and erythropoietin injections [60]. All studies measured muscle quantity (DXA, CT or BIA) and both GH trials measured muscle strength. The only study that measured physical performance was the multi-arm study including physical activity and nutritional interventions [38]. One GH trial showed statistically significant amelioration in muscle quantity loss by DXA [32] and the other showed statistically significant amelioration in knee flexion strength loss [31]. Adverse events were similar between control and intervention arms [31]; one study showed slightly higher peripheral oedema rates amongst GH recipients [32]. The nandrolone trial did not report statistically significant results [33]. Erythropoietin induced a small statistically significant amelioration in muscle quantity loss after orthopaedic surgery, not related to haemoglobin changes. Testosterone was safe in the multi-arm study, with statistically significant amelioration in physical performance demonstrated comparing all intervention groups to placebo [38].

### Neuromuscular electrical stimulation

Three trials involved NMES [34, 35, 52]; all combined NMES with exercise. One trial (geriatric medicine population) tested functional training alone against functional training with NMES [34]. No statistically significant different change in gait speed between groups was demonstrated. Another trial (respiratory medicine population) showed significant lesser decline in knee extension strength with NMES [35]. The third trial (general medicine population) resulted in significant improvements in physical performance with NMES [52].

### Risk of bias and certainty across studies

**Figure 2:**
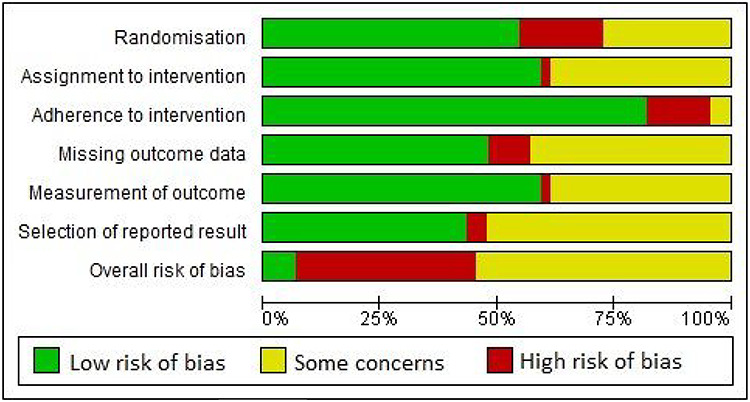
Risk of bias results across all included studies.

Figure 2 shows overall risk of bias across studies. Full risk of bias details is shown in the Supplementary Appendix. There were at least some concerns for overall risk of bias across most studies. Adherence to trial intervention was associated with lowest risk and selection of reported outcome with highest risk. Most common reason for high risk of bias related to randomisation processes. Over half of studies exhibited at least some concerns for selection of reported result. Table 3 shows assessment of certainty for two interventions (individualised physical training programmes and protein supplementation) across studies.

**Table 3:** GRADE domain certainty for individual physical training programmes and protein supplementation

GRADE Domain	Certainty	Comments
Individual physical training programme		
Risk of bias	Moderate	RCTs assessed were mainly considered to have some concerns for overall risk of bias; no studies with high risk of bias.
Imprecision	Moderate	Meta-analysis of effect sizes across RCTs was not performed, although larger sample sizes in included studies.
Inconsistency	Low	Inconsistency of effect sizes across studies.
Indirectness	High	All but one study in geriatric medicine setting; all in older adults. All patients able to ambulate pre-admission and at risk of functional decline.
Publication bias	High	Publication bias of RCTs unlikely, particularly as mixed results presented. Inclusion of thesis and conference abstract for another physical activity intervention included.
Protein supplementation (with or without exercise)		
Risk of bias	Moderate	RCTs assessed were considered to have either low risk or some concerns for overall risk of bias.
Imprecision	Low	Overall small number of studies with low sample sizes.
Inconsistency	Moderate	Similar effect sizes demonstrated in small numbers of studies.
Indirectness	High	All studies performed in general/geriatric medicine setting in older adults.
Publication bias	High	Publication bias of RCTs unlikely, particularly considering identification of studies with low sample size.

## Discussion

### Interpretation of findings

Physical activity interventions were investigated more commonly than others. However, this mostly relates to studies with physical performance outcomes; only four trials not involving physical activity interventions measured physical performance [26, 39, 48, 49]. Conversely, many physical activity trials reported muscle strength change but only one measured muscle quantity change, a multi-arm study also involving nutritional/pharmaceutical interventions. Nutritional and pharmaceutical trials focused on muscle strength and quantity changes rather than physical performance. This suggests disconnect in how physical activity interventions are trialled compared to other interventions; physical performance declines may not be prioritised as organ insufficiency markers in need of urgent treatment.

Only nine trials reported muscle quantity change. This relates to historical reduced availability of feasible serial assessment tools; DXA, CT and MRI remain gold-standard, but ultrasound is increasingly utilised [1,16]. As sarcopenia definition has developed, measures of muscle function are considered more important than muscle quantity [1]. However, in acute sarcopenia, early muscle quantity declines may not be associated with muscle strength declines [3]; preventing this may be important to prevent longer term deteriorations. Additionally, muscle strength may be affected by fatigue/effort during acute illness making testing of efficacy/effectiveness challenging [17]. Muscle quantity may be an appropriate treatment target in hospitalised patients; future trials of interventions for acute sarcopenia should consider incorporating in outcomes. Measurement of muscle quantity is also important to show biological effectiveness/mechanistic action.

We identified several physical activity interventions that stratified treatment protocols individually (e.g. by frailty) [37, 43, 45, 53, 55]. Most substantial and significant effects on muscle strength and physical performance were demonstrated in the highest reported adherence trial [43]. Although this demonstrates high adherence of hospitalised older adults to complex trial designs is possible, effectiveness is expected to be reduced in clinical environments with limited compliance. Increasing mobilisation alone may be insufficient to prevent/treat acute sarcopenia [17, 20, 22, 24, 41], although this is safe to do when possible and should be commended [17, 20, 24, 25, 41]. Physical activity interventions can be multidimensional and include resistance exercise [43, 44]; it is safe and feasible to use machines/weights during acute phase of illness in hospitalised older patients [43, 44, 57]. Pedal exercises [23, 36] and seated side-tapping [47] are simple, cheap, feasible and potentially effective; these may be implemented as part of multidimensional stratified interventions. Group exercise may be as effective as individual exercise but more cost-effective [42]. Group exercise has additional benefits of improving social interaction, and potentially improving motivation [18] and adherence [43].

Several nutritional interventions were trialled. Although few trials showed statistically significant results, all trials were small and may have been under-powered for efficacy. Three trials combined nutritional intervention with physical activity [27, 38, 51]. Research in chronic sarcopenia suggested additional protein supplementation may be most effective when combined with targeted physical activity i.e. resistance exercise [19]. As inflammation and anabolic resistance are heightened with acute illness [3], greater doses (i.e. greater protein/amino acid intake) may be warranted in hospitalised older adults.

Few studies tested pharmaceuticals. There is suggestion from GH trials that this may be effective in ameliorating reductions in muscle quantity and strength [31, 32]. Further research is needed, including longer term outcomes. Benefits of GH supplementation need to be balanced against adverse effects, although supplementation was safe in dosages used in these small studies. Research is ongoing into novel pharmaceutical agents for use in acute and chronic sarcopenia [20]. Studies assessing correlations between immune-endocrine biomarkers and phenotypic changes in muscle quantity, quality or function will enable stratified treatments and direct potential drug pathways.

Trials of NMES showed conflicting results. NMES involves delivery of controlled electrical stimuli to superficial muscles via self-adhesive skin electrodes. These stimuli evoke muscle contractions, recruiting motor units and activating muscle fibres [21]. NMES has been shown to ameliorate reductions in muscle quantity and function in healthy young volunteers during bed rest [22]. It is plausible that NMES may treat acute sarcopenia in hospitalised older adults. However, in establishing effectiveness in clinical practice, adherence, physical activity impact, and which muscle groups to stimulate should be considered.

### What are the limitations of this review?

This review included hospitalised adults over 65 years-old. We excluded younger adults to focus towards most vulnerable patients, who are most likely to benefit from targeted interventions. More studies were excluded for participant age than were included (56 versus 44). This suggests persistent bias against involvement of older people in clinical trials, particularly those with frailty. Considering we included search terms for older people in our search, it is likely more trials involving younger adults were not identified, as well as trials excluded through abstract screening. Trials conducted in younger adults may be useful when developing interventions for acute sarcopenia in older adults, but caution should be taken extrapolating results from younger less heterogeneous populations.

It is important to consider only three studies reported frailty status in both control and intervention arms [37, 45, 50]. Frailty was measured in intervention arms but rates were not reported in studies that stratified by frailty [55, 57]. Although important measures, handgrip strength and gait speed alone may be insufficient to diagnose pre-morbid frailty during acute illness [42]. Recording levels of frailty prior to hospitalisation can ensure control and intervention arms are matched and enable sub-group analysis assessing treatment effect in individuals with and without frailty [45]. Only one study reported ethnicity amongst participants [38]. Normative values of muscle quantity may vary according to ethnicity [23], and muscle echotexture may differ [24]. Further research is needed to assess effects of genetics and environment on ethnic differences, and how these relate to differences in muscle function and responsiveness to interventions. Without information on ethnicity within published trials, it is not possible to assess for between group differences.

As described, majority of trials were small; many may have been underpowered to detect changes. Due to high heterogeneity in populations, interventions, and outcome measures, it was not possible to conduct meta-analyses. Some interventions that were not shown to be effective in small individual trials may be effective in larger powered studies. Additionally, most studies exhibited some concerns for risk of bias overall, and due to inconsistencies in effect sizes across different studies, there is limited certainty around true effect sizes. Many different outcome measures were also assessed across different RCTs. We consider that standardisation of assessment and outcome measures within geriatric medicine research will enable greater ease of knowledge transfer, sharing of datasets and future meta-analyses of RCTs in ageing.

It is important to consider that none of the included trials specifically included the presence of (acute or chronic) sarcopenia as inclusion criteria, or stratified treatment by sarcopenia. However, we consider that results of identified RCTs identified will be pivotal towards designing trials for prevention and/or treatment of acute sarcopenia. Acute sarcopenia is a rapidly progressing research area and therapeutic target. Twenty-two percent of studies (10/44) included in this review were published in the last 18 months. This demonstrates how rapidly progressive this area is, with increasing numbers of studies measuring muscle quantity and function as outcome measures.

## Conclusion

Deteriorations in muscle quantity, strength and physical performance are problematic in older adults following hospitalisation. However, insufficient evidence exists to enable targeted prevention/treatment strategies. A number of interventions have been trialled and shown to be safe for heterogeneous populations across various settings. Multidimensional physical activity interventions which are individually tailored (e.g. for frailty) have been trialled [43, 45, 55]; the trial with most substantial effect size reported excellent adherence [43]. Large scale multi-arm studies assessing effectiveness of combined interventions including physical activity [23, 43, 45, 47, 55], NMES [34], nutrition [59] and pharmaceuticals [31, 32] are warranted. Treatment may be most effective when stratified according to individual need. Treatment is likely to be guided by a combination of clinical and biological factors (e.g. immune-endocrine markers). Further research aimed at understanding pathophysiology of acute sarcopenia will enable risk stratification and targeted interventions.

## Supplementary Material

aa-20-0102-File002_afaa209
