# Supplementary material for: Interventions to ameliorate reductions in muscle quantity and function in hospitalised older adults: a systematic review towards acute sarcopenia treatment
Source: Age Ageing. 2020 Oct 24;50(2):394–404. doi: 10.1093/ageing/afaa209 (PMC7936029; doi:10.1093/ageing/afaa209)
Supplement: aa-20-0102-File002_afaa209 [file aa-20-0102-file002_afaa209.docx]

# Supplementary Data

# Interventions to ameliorate reductions in muscle quantity and function in hospitalised older adults: a systematic review towards acute sarcopenia treatment

## Full Reference List

1. Cruz-Jentoft AJ, Bahat G, Bauer J, Boirie Y, Bruyere O, Cederholm T, et al. Sarcopenia: revised European consensus on definition and diagnosis. Age Ageing. 2018.

2. Cruz-Jentoft AJ, Sayer AA. Sarcopenia. The Lancet. 2019;393(10191):2636-46.

3. Welch C, Hassan-Smith Z, Greig C, Lord J, Jackson T. Acute Sarcopenia Secondary to Hospitalisation - An Emerging Condition Affecting Older Adults. Aging and Disease. 2018;9(1):151-64.

4. Cruz-Jentoft AJ, Bahat G, Bauer J, Boirie Y, Bruyère O, Cederholm T, et al. Sarcopenia: revised European consensus on definition and diagnosis. Age and Ageing. 2018:afy169-afy.

5. Yoshimura Y, Wakabayashi H, Yamada M, Kim H, Harada A, Arai H. Interventions for Treating Sarcopenia: A Systematic Review and Meta-Analysis of Randomized Controlled Studies. Journal of the American Medical Directors Association. 2017;18(6):553.e1-.e16.

6. Beaudart C, Dawson A, Shaw SC, Harvey NC, Kanis JA, Binkley N, et al. Nutrition and physical activity in the prevention and treatment of sarcopenia: systematic review. Osteoporosis international : a journal established as result of cooperation between the European Foundation for Osteoporosis and the National Osteoporosis Foundation of the USA. 2017;28(6):1817-33.

7. Cruz-Jentoft AJ, Landi F, Schneider SM, Zúñiga C, Arai H, Boirie Y, et al. Prevalence of and interventions for sarcopenia in ageing adults: a systematic review. Report of the International Sarcopenia Initiative (EWGSOP and IWGS). Age and Ageing. 2014;43(6):748-59.

8. Beckwee D, Delaere A, Aelbrecht S, Baert V, Beaudart C, Bruyere O, et al. Exercise Interventions for the Prevention and Treatment of Sarcopenia. A Systematic Umbrella Review. J Nutr Health Aging. 2019;23(6):494-502.

9. Denison HJ, Cooper C, Avan AS, Robinson SM. Prevention and optimal management of sarcopenia: A review of combined exercise and nutrition interventions to improve muscle outcomes in older people. Clinical Interventions in Aging. 2015;10:859-69.

10. Marzetti E. The SPRINTT project: tackling physical frailty and sarcopenia to prevent disability in the elderly. Free radical biology and medicine Conference: 19th biennial meeting for the society for free radical research international, SFRRI 2018 Portugal. 2018;120(Supplement 1):S16.

11. Morton RW, Traylor DA, Weijs PJM, Phillips SM. Defining anabolic resistance: implications for delivery of clinical care nutrition. Current opinion in critical care. 2018;24(2):124-30.

12. The Cochrane Collaboration. Review Manager (RevMan). 5.3 ed. Copenhagen: The Nordic Cochrane Centre The Cochrane Collaboration; 2014.

13. Siemieniuk RG, Gordon. What is GRADE? : BMJ Best Practice; [Available from: <https://bestpractice.bmj.com/info/toolkit/learn-ebm/what-is-grade/>.

14. The Cochrane Collaboration. Imputing standard deviations for changes from baseline. In: J.P.T Higgins SG, editor. Cochrane Handbook for Systematic Reviews of Interventions. 5.1.0 ed2011.

15. The Cochrane Collaboration. The standardized mean difference. In: J.P.T Higgins SG, editor. Cochrane Handbook for Systematic Reviews of Interventions. 5.1.0 ed2011.

16. Cohen J. A power primer. Psychological Bulletin. 1992;112(1):155-9.

17. Wnuk BR, Durmala J, Ziaja K, Kotyla P, Wozniewski M, Blaszczak E. A Controlled Trial of the Efficacy of a Training Walking Program in Patients Recovering from Abdominal Aortic Aneurysm Surgery. Advances in clinical and experimental medicine : official organ Wroclaw Medical University. 2016;25(6):1241-371.

18. Rahmann AE, Brauer SG, Nitz JC. A specific inpatient aquatic physiotherapy program improves strength after total hip or knee replacement surgery: a randomized controlled trial. Archives of Physical Medicine & Rehabilitation. 2009;90(5):745-55.

19. Giangregorio LM, Thabane L, DeBeer J, Farrauto L, McCartney N, Adachi JD, et al. Body weight-supported treadmill training for patients with hip fracture: a feasibility study. Archives of Physical Medicine & Rehabilitation. 2009;90(12):2125-30.

20. Fiore JF, Jr., Castelino T, Pecorelli N, Niculiseanu P, Balvardi S, Hershorn O, et al. Ensuring Early Mobilization Within an Enhanced Recovery Program for Colorectal Surgery: A Randomized Controlled Trial. Ann Surg. 2017;266(2):223-31.

21. Tal-Akabi A, Steiger U, Villiger PM. Neuromuscular adaptation to early post-operative, high-intensity, short resistance training of non-operated lower extremity in elderly patients: a randomized controlled trial. Journal of Rehabilitation Medicine (Stiftelsen Rehabiliteringsinformation). 2007;39(9):724-9.

22. Said CM, Morris ME, Woodward M, Churilov L, Bernhardt J. Enhancing physical activity in older adults receiving hospital based rehabilitation: a phase II feasibility study. BMC geriatrics. 2012;12:26.

23. Torres-Sanchez I, Valenza MC, Cabrera-Martos I, Lopez-Torres I, Benitez-Feliponi A, Conde-Valero A. Effects of an Exercise Intervention in Frail Older Patients with Chronic Obstructive Pulmonary Disease Hospitalized due to an Exacerbation: a Randomized Controlled Trial. COPD: journal of chronic obstructive pulmonary disease. 2017;14(1):37‐42.

24. Blanc-Bisson C, Dechamps A, Gouspillou G, Dehail P, Bourdel-Marchasson I. A randomized controlled trial on early physiotherapy intervention versus usual care in acute care unit for elderly: potential benefits in light of dietary intakes. Journal of nutrition, health & aging. 2008;12(6):395‐9.

25. Henriksen MG, Jensen MB, Hansen HV, Jespersen TW, Hessov I. Enforced mobilization, early oral feeding, and balanced analgesia improve convalescence after colorectal surgery. Nutrition. 2002;18(2):147-52.

26. Niccoli S, Kolobov A, Bon T, Rafilovich S, Munro H, Tanner K, et al. Whey Protein Supplementation Improves Rehabilitation Outcomes in Hospitalized Geriatric Patients: a Double Blinded, Randomized Controlled Trial. Journal of nutrition in gerontology and geriatrics. 2017;36(4):149‐65.

27. Hermanky M, Korninger C, Fuchs D, Strasser B. Effects of a protein-optimized diet in combination with moderate strength training on the postoperative course in elderly patients with hip fracture. Aktuel Ernahrungsmed. 2017;42(03):180-7.

28. Saudny-Unterberger H, Martin JG, Gray-Donald K. Impact of nutritional support on functional status during an acute exacerbation of chronic obstructive pulmonary disease. Am J Respir Crit Care Med. 1997;156(3 Pt 1):794-9.

29. Ogasawara T, Marui S, Miura E, Sugiura M, Matsuyama W, Aoshima Y, et al. Effect of eicosapentaenoic acid on prevention of lean body mass depletion in patients with exacerbation of chronic obstructive pulmonary disease: A prospective randomized controlled trial. Clinical nutrition ESPEN. 2018;28:67-73.

30. Bouillanne O, Melchior JC, Faure C, Paul M, Canoui-Poitrine F, Boirie Y, et al. Impact of 3-week citrulline supplementation on postprandial protein metabolism in malnourished older patients: the Ciproage randomized controlled trial. Clinical nutrition (Edinburgh, Scotland). 2018;(no pagination).

31. Weissberger AJ, Anastasiadis AD, Sturgess I, Martin FC, Smith MA, Sönksen PH. Recombinant human growth hormone treatment in elderly patients undergoing elective total hip replacement. Clinical endocrinology. 2003;58(1):99‐107.

32. Hedström M, Sääf M, Brosjö E, Hurtig C, Sjöberg K, Wesslau A, et al. Positive effects of short-term growth hormone treatment on lean body mass and BMC after a hip fracture: a double-blind placebo-controlled pilot study in 20 patients. Acta orthopaedica scandinavica. 2004;75(4):394‐401.

33. Sloan JP, Wing P, Dian L, Meneilly GS. A pilot study of anabolic steroids in elderly patients with hip fractures. Journal of the american geriatrics society. 1992;40(11):1105‐11.

34. Zinglersen AH, Halsteen MB, Kjaer M, Karlsen A. Can electrical stimulation enhance effects of a functional training program in hospitalized geriatric patients? Experimental gerontology. 2018;106:101‐8.

35. Martin-Salvador A, Colodro-Amores G, Torres-Sanchez I, Moreno-Ramirez MP, Cabrera-Martos I, Valenza MC. Physical therapy intervention during hospitalization in patients with acute exacerbation of chronic obstructive pulmonary disease and pneumonia: A randomized clinical trial. Medicina clinica. 2016;146(7):301-4.

36. McGowan T, Ong T, Kumar A, Lunt E, Sahota O. The effect of chair-based pedal exercises for older people admitted to an acute hospital compared to standard care: a feasibility study. Age & Ageing. 2018;47(3):483-6.

37. Braun T, Grüneberg C, Süßmilch K, Wiessmeier M, Schwenk I, Eggert S, et al. An augmented prescribed exercise program (APEP) to improve mobility of older acute medical patients – a randomized, controlled pilot and feasibility trial. BMC Geriatrics. 2019;19(1):240.

38. Deer RR, Dickinson JM, Baillargeon J, Fisher SR, Raji M, Volpi E. A Phase I Randomized Clinical Trial of Evidence-Based, Pragmatic Interventions to Improve Functional Recovery After Hospitalization in Geriatric Patients. The Journals of Gerontology: Series A. 2019;74(10):1628-36.

39. Files DC, Heinrich T, Shields KL, Love NJ, Brailer C, Bakhru RN, et al. A randomized pilot study of nitrate supplementation with beetroot juice in acute respiratory failure. Nitric Oxide. 2020;94:63-8.

40. Prasciene E, Icking N, Kisieliute E, Kubilius R, Bjarnason-Wehrens B. Feasibility and short-term effectiveness of an additional resistance and balance training in cardiac rehabilitation for older patients after valve surgery: results of a pilot study. EuroPrevent 2019; Portugal: European Journal of Preventive Cardiology; 2019. p. S39.

41. de Morton NA, Keating JL, Berlowitz DJ, Jackson B, Lim WK. Additional exercise does not change hospital or patient outcomes in older medical patients: a controlled clinical trial. Australian Journal of Physiotherapy. 2007;53(2):105-11.

42. Raymond MJ, Jeffs KJ, Winter A, Soh SE, Hunter P, Holland AE. The effects of a high-intensity functional exercise group on clinical outcomes in hospitalised older adults: an assessor-blinded, randomised-controlled trial. Age and ageing. 2017;46(2):208‐13.

43. Martínez-Velilla N, Casas-Herrero A, Zambom-Ferraresi F, Sáez de Asteasu ML, Lucia A, Galbete A, et al. Effect of Exercise Intervention on Functional Decline in Very Elderly Patients During Acute Hospitalization: A Randomized Clinical Trial. JAMA internal medicine. 2019;179(1):28-36.

44. Busch JC, Lillou D, Wittig G, Bartsch P, Willemsen D, Oldridge N, et al. Resistance and balance training improves functional capacity in very old participants attending cardiac rehabilitation after coronary bypass surgery. Journal of the american geriatrics society. 2012;60(12):2270‐6.

45. McCullagh R. A randomised controlled trial to measure the effects of an augmented prescribed exercise programme (APEP) on length of stay, physical ability and quality of life in frail older medical patients in the acute setting: University of Cork; 2017.

46. Houborg KB, Jensen MB, Hessov I, Laurberg S. Little effect of physical training on body composition and nutritional intake following colorectal surgery--a randomised placebo-controlled trial. Eur J Clin Nutr. 2005;59(8):969-77.

47. Sano YI, A. Wanaka, H. Matsui, M. Yamamoto, S. Koyanagi, J. Iwata, H. An easy and safe training method for trunk function improves mobility in total knee arthroplasty patients: a quasi-randomized controlled trial. Plos one. 2018;13(10) (no pagination).

48. Beelen J, de Roos NM, de Groot L. A 12-week intervention with protein-enriched foods and drinks improved protein intake but not physical performance of older patients during the first 6 months after hospital release: a randomised controlled trial. British journal of nutrition. 2017;117(11):1541‐9.

49. Gade J, Beck AM, Andersen HE, Christensen B, Rønholt F, Klausen TW, et al. Protein supplementation combined with low-intensity resistance training in geriatric medical patients during and after hospitalisation: a randomised, double-blind, multicentre trial. The British journal of nutrition. 2019;122(9):1006-20.

50. Ortiz-Alonso J, Bustamante-Ara N, Valenzuela PL, Vidán-Astiz M, Rodríguez-Romo G, Mayordomo-Cava J, et al. Effect of a Simple Exercise Program on Hospitalization-Associated Disability in Older Patients: A Randomized Controlled Trial. Journal of the American Medical Directors Association. 2020;21(4):531-7.e1.

51. Pedersen MM, Petersen J, Beyer N, Larsen HG, Jensen PS, Andersen O, et al. A randomized controlled trial of the effect of supervised progressive cross-continuum strength training and protein supplementation in older medical patients: the STAND-Cph trial. Trials. 2019;20(1):655.

52. López-López L, Torres-Sánchez I, Rodríguez-Torres J, Cabrera-Martos I, Ortiz-Rubio A, Valenza MC. Does adding an integrated physical therapy and neuromuscular electrical stimulation therapy to standard rehabilitation improve functional outcome in elderly patients with pneumonia? A randomised controlled trial. Clin Rehabil. 2019;33(11):1757-66.

53. Jones CT, Lowe AJ, MacGregor L, Brand CA, Tweddle N, Russell DM. A randomised controlled trial of an exercise intervention to reduce functional decline and health service utilisation in the hospitalised elderly. Australasian Journal on Ageing. 2006;25(3):126-33.

54. Moseley AM, Sherrington C, Lord SR, Barraclough E, St George RJ, Cameron ID. Mobility training after hip fracture: a randomised controlled trial. Age Ageing. 2009;38(1):74-80.

55. Opasich C, Patrignani A, Mazza A, Gualco A, Cobelli F, Pinna GD. An elderly-centered, personalized, physiotherapy program early after cardiac surgery. European journal of cardiovascular prevention and rehabilitation : official journal of the European Society of Cardiology, Working Groups on Epidemiology & Prevention and Cardiac Rehabilitation and Exercise Physiology. 2010;17(5):582-7.

56. Said CM, Morris ME, McGinley JL, Szoeke C, Workman B, Liew D, et al. Additional structured physical activity does not improve walking in older people (> 60 years) undergoing inpatient rehabilitation: a randomised trial. Journal of Physiotherapy (Elsevier). 2018;64(4):237-44.

57. Schwenk M, Dutzi I, Englert S, Micol W, Najafi B, Mohler J, et al. An intensive exercise program improves motor performances in patients with dementia: translational model of geriatric rehabilitation. Journal of Alzheimer's disease : JAD. 2014;39(3):487-98.

58. Sherrington C, Lord SR, Herbert RD. A randomised trial of weight-bearing versus non-weight-bearing exercise for improving physical ability in inpatients after hip fracture. Australian Journal of Physiotherapy. 2003;49(1):15-22.

59. Ekinci O, Yanık S, Terzioğlu Bebitoğlu B, Yılmaz Akyüz E, Dokuyucu A, Erdem Ş. Effect of Calcium β-Hydroxy-β-Methylbutyrate (CaHMB), Vitamin D, and Protein Supplementation on Postoperative Immobilization in Malnourished Older Adult Patients With Hip Fracture. Nutrition in Clinical Practice. 2016;31(6):829-35.

60. Zhang Y, Chen L, Wu P, Lang J, Chen L. Intervention with erythropoietin in sarcopenic patients with femoral intertrochanteric fracture and its potential effects on postoperative rehabilitation. Geriatrics & Gerontology International. 2020;20(2):150-5.

61. Wilson DV, Moorey H, Stringer H, Sahbudin I, Filer A, Lord JM, et al. Bilateral Anterior Thigh Thickness: A New Diagnostic Tool for the Identification of Low Muscle Mass? Journal of the American Medical Directors Association. 2019.

62. Van Ancum JM, Scheerman K, Jonkman NH, Smeenk HE, Kruizinga RC, Meskers CGM, et al. Change in muscle strength and muscle mass in older hospitalized patients: A systematic review and meta-analysis. Experimental gerontology. 2017;92:34-41.

63. Fuller LM, Button B, Tarrant B, Battistuzzo CR, Braithwaite M, Snell G, et al. Patients' expectations and experiences of rehabilitation following lung transplantation. Clinical transplantation. 2014;28(2):252-8.

64. Martone AM, Marzetti E, Calvani R, Picca A, Tosato M, Santoro L, et al. Exercise and Protein Intake: A Synergistic Approach against Sarcopenia. Biomed Res Int. 2017;2017:2672435-.

65. Hardee JP, Lynch GS. Current pharmacotherapies for sarcopenia. Expert Opinion on Pharmacotherapy. 2019;20(13):1645-57.

66. Maffiuletti NA, Green DA, Vaz MA, Dirks ML. Neuromuscular Electrical Stimulation as a Potential Countermeasure for Skeletal Muscle Atrophy and Weakness During Human Spaceflight. Front Physiol. 2019;10(1031).

67. Dirks ML, Wall BT, Snijders T, Ottenbros CLP, Verdijk LB, van Loon LJC. Neuromuscular electrical stimulation prevents muscle disuse atrophy during leg immobilization in humans. Acta Physiologica. 2014;210(3):628-41.

68. Silva AM, Shen W, Heo M, Gallagher D, Wang Z, Sardinha LB, et al. Ethnicity-related skeletal muscle differences across the lifespan. Am J Hum Biol. 2010;22(1):76-82.

69. Melvin MN, Smith-Ryan AE, Wingfield HL, Fultz SN, Roelofs EJ. Evaluation of muscle quality reliability and racial differences in body composition of overweight individuals. Ultrasound Med Biol. 2014;40(9):1973-9.

## Appendix 1 – Search strategy

|  | Search terms |
| --- | --- |
| MEDLINE | 1. randomized controlled trial.pt OR controlled clinical trial.pt OR randomized.ti,ab OR randomised.ti,ab OR placebo.ti,ab OR drug therapy.hw OR randomly.ti,ab OR trial.ti,ab OR groups.ti,ab  2. Humans.sh  3. 1 AND 2  4. "Aged".sh OR "Aged, 80 and over".sh OR "Frail Elderly".sh  5. elder*.ti,ab OR septuagenarian*.ti,ab OR octogenarian*.ti,ab OR nonagenarian*.ti,ab OR centenarian*.ti,ab OR older.ti,ab OR geriatric*.ti,ab  6. 4 OR 5  7. "Hospitalization".sh OR "Patient Admission".sh  8. hospital*.ti,ab OR admission*.ti,ab OR inpatient*.ti,ab OR admitted.ti,ab  9. 7 OR 8  10. "Muscular Atrophy".sh  11. (musc* ADJ2 mass).ti,ab OR (musc* ADJ2 size).ti,ab OR (musc* ADJ2 atroph*).ti,ab OR (musc* ADJ2 wast*).ti,ab OR (musc* ADJ2 loss*).ti,ab  12. 10 OR 11  13. "Muscle Weakness".sh  14. (musc* ADJ2 weak*).ti,ab OR (musc* ADJ2 strength).ti,ab OR (musc ADJ2 strong*).ti,ab  15. 13 OR 14  16. “Mobility limitation”.sh  17. (speed* ADJ2 gait).ti,ab OR (walk* ADJ2 speed*).ti,ab OR (physical ADJ performance).ti,ab OR ambulat*.ti,ab or mobil*.ti,ab  18. 16 OR 17  19. 3 AND 6 AND 9  20. 12 OR 15 OR 18  21. 19 AND 20 |
| EMBASE | 1. (random* OR factorial* OR crossover* OR cross over* OR cross-over* OR placebo* OR doubl* blind* OR singl* blind* OR assign* OR allocat* OR volunteer*).af.  2. exp crossover-procedure/ or exp double-blind procedure/ or exp randomized controlled trial/ or exp single-blind procedure/  3. 1 OR 2  4. (exp geriatric patient/ or exp aged/).ti,ab  5. (elder* OR septuagenarian* OR octogenarian* OR nonagenarian* OR centenarian* OR older OR geriatric*).ti,ab  6. 4 OR 5  7. exp hospitalization/ or exp hospital patient/ or exp hospital admission  8. (hospital* OR admission* OR inpatient* OR admitted).ti,ab  9. 7 OR 8  10. exp muscle atrophy/ or exp muscle mass/  11. ((musc* ADJ2 mass) OR (musc* ADJ2 size) OR (musc* ADJ2 atroph*) OR (musc* ADJ2 wast*) OR (musc* ADJ2 loss*)).ti,ab  12. 10 OR 11  13. exp muscle weakness/ or exp muscle strength/  14. ((musc* ADJ2 weak*) OR (musc* ADJ2 strength) OR (musc ADJ2 strong*)).ti,ab  15. 13 OR 14  16. exp physical performance/ or exp walking speed/  17. ((speed* ADJ2 gait) OR (walk* ADJ2 speed*) OR (physical ADJ performance) OR (ambulat* OR mobil*)).ti,ab  18. 16 OR 17  19. 3 AND 6 AND 9  20. 12 AND 15 AND 18  21. 19 AND 20 |
| CINAHL | 1. (MH “Randomized controlled trials”) 2. RCT OR randomised OR randomized OR random OR placebo OR trial OR crossover OR masked OR blind 3. 1 OR 2 4. (MH “Aged”) OR (MH “Aged, 80 and Over) OR (MH Aged, Hospitalized) OR (MH Frail Elderly) 5. elder* OR septuagenarian* OR octogenarian* OR nonagenarian* OR centenarian* OR older OR geriatric* 6. 4 or 5 7. (MH “Hospitalization”) OR (MH “Patient Admission”) 8. hospital* OR admission* OR inpatient* OR admitted 9. 7 OR 8 10. (MH “Muscular Atrophy”) 11. (musc* N2 mass) OR (musc* N2 size) OR (musc* N2 atroph*) OR (musc* N2 wast*) OR (musc* N2 loss*) 12. 10 OR 11 13. (MH “Muscle weakness”) OR (MH “Muscle strength”) OR (MH “Grip strength”) 14. (musc* N2 weak*) OR (musc* N2 strength) OR (musc N2 strong*) 15. 13 OR 14 16. (MH “Physical Performance) 17. (speed* N2 gait) OR (walk* N2 speed*) OR (physical performance) OR ambulat* OR mobil* 18. 16 OR 17 19. 3 AND 6 AND 9 20. 12 OR 15 OR 18 21. 19 AND 20 |
| Cochrane Library (CENTRAL) | 1. [mh "Aged"] OR [mh "Aged, 80 and over"] OR [mh "Frail Elderly"]  2. elder* OR septuagenarian* OR octogenarian* OR nonagenarian*OR centenarian* OR older OR geriatric*  3. 1 OR 2  4. [mh "Hospitalization"] OR [mh "Patient Admission"]  5. hospital* OR admission* OR inpatient* OR admitted  6. 4 OR 5  7. [mh "Muscular Atrophy"]  8. musc* NEAR/2 mass OR musc* ADJ2 size OR musc* NEAR/2 atroph* OR musc* NEAR/2 wast* OR musc* NEAR/2 loss*  9. 7 OR 8  10. [mh "Muscle Weakness"]  11. musc* NEAR/2 weak* OR musc* NEAR/2 strength OR musc NEAR/2 strong*  12. 10 OR 11  13. [mh "Mobility limitation"]  14. speed* NEAR/2 gait OR walk* NEAR/2 speed* OR physical performance OR ambulat* or mobil*  15. 13 OR 14  16. 3 AND 6  17. 9 OR 12 OR 15  18. 16 AND 17 |

## Appendix 2 – Full study characteristics and results

| Intervention type | Outcome type | Study  1. First author  2. Year  3. Country | Setting  1. Specialty  2. Participant numbers | Participants: comparison  1. Age (years (SD))  2. Gender (%Female)  3. BMI (mean, SD) | Participants: intervention  1. Age (years (SD))  2. Gender (%Female)  3. BMI (mean, SD) | Comparison  Usual care or placebo | Measures and timing  1. Measure  2. Baseline timing  3. Post-intervention | Comparison outcomes | | Intervention  1. Name  2. Regimen, duration, how delivered | Intervention outcomes | | SMD of ∆ | Between group and time statistical significance |
| --- | --- | --- | --- | --- | --- | --- | --- | --- | --- | --- | --- | --- | --- | --- |
|  |  |  |  |  |  |  |  | 1. Baseline (mean, SD)  2. Post-intervention (mean, SD) | 3. Mean change, SD  4. Within group statistical significance |  | 1. Baseline (mean, SD)  2. Post-intervention (mean, SD) | 3. Mean change, SD  4. Within group statistical significance |  |  |
| Physical activity | Physical performance | 1.Wnuk(1)  2. 2016  3. Poland | 1. Vascular  2. Control n=16 Intervention n=15 | 1. 69 (4)  2. 0  3. 26.3 (3.5) | 1. 68 (3)  2. 0  3. 26.2 (3.5) | Usual care (basic physiotherapy) | 1. 6MWT (m)  2. Pre-operative 3. One week postoperative | 1. 324.2 (63.4)  2. 258.1 (60.4) | 3. -66.1 (45.6) | 1. Backward walking  2. Steadily increased frequency for first 3 days then increased duration. | 1. 362.3 (41.7)  2. 322.4 (64.7) | 3. -39.9 | +0.6 | p=0.029  **Favours experimental** |
|  |  | 1.Wnuk (1)  2. 2016  3. Poland | 1. Vascular  2. Control n=16 Intervention n=16 | 1. 69 (4)  2. 0  3. 26.3 (3.5) | 1. 70 (3)  2. 0  3. 26.6 (2.5) | Usual care (basic physiotherapy) | 1. 6MWT (m)  2. Pre-operative 3. One week postoperative | 1. 324.2 (63.4)  2. 258.1 (60.4) | 3. -66.1 (45.6) | 1. Forward walking  2. Steadily increased frequency for first 3 days then increased duration. | 1. 338.3 (70.8)  2. 304.3 (73.0) | 3. -34.0 | +0.7 | p=0.130  Favours null |
|  |  | 1. Sherrington (2)  2. 2003  3. Australia | 1. Orthopaedic rehabilitation  2. Control n=39  Intervention n=41 | 1. 81.1 (8.3)  2. 69  3. Unknown | 1. 81.0 (7.0)  2. 66  3. Unknown | Non-weight-bearing exercise | 1. Gait speed (m/s)  2. After randomisation  3. Two weeks after baseline | 1. 0.09 (0.09)  2. 0.19 (0.20) | 3. +0.10 (0.17) | 1. Weight-bearing exercise  2. Individually progressed stepping exercises – increased repetitions, lessening hand support, increasing height of blocks. | 1. 0.12 (0.10)  2. 0.25 (0.22) | 3. +0.13 | +0.2 | p=0.69  Favours null |
|  |  | 1. Rahmann (3)  2. 2009  3. Australia | 1. Elective orthopaedic  2. Control n=20  Intervention n=24 | 1. 70.4 (9.2)  2. 70.6  3 28.8 (6.2) | 1. 69.4 (6.5)  2. 44.4  3. 28.4 (4.6) | Ward-based physiotherapy | 1. TUG (s)  2. Pre-operative  3. Two weeks postoperative | 1. 15.5 (6.8)  2. 25.4 (14.4) | 3. +9.9 (9.7) | 1. Aquatic physiotherapy  2. Progressive trunk stability, backstroke kick, arm swing exercises in water | 1. 12.3 (3.3)  2. 18.4 (10.1) | 3. +6.2 | +0.4 | p=0.092  Favours null |
|  |  | 1. Rahmann (3)  2. 2009  3. Australia | 1. Elective orthopaedic  2. Control n=24  Intervention n=21 | 1. 69.4 (6.5)  2. 44.4  3. 28.4 (4.6) | 1. 69.0 (8.9)  2. 43.2  3. 28.0 (4.1) | Aquatic physiotherapy | 1. TUG (s)  2. Pre-operative  3. Two weeks postoperative | 1. 12.3 (3.3)  2. 18.4 (10.1) | 3. +6.2 (7.5) | 1. Water exercise  2. Progressive trunk stability, backstroke kick, arm swing exercises in water | 1. 16.4 (9.7)  2. 19.7 (6.7) | 3. +3.3 | +0.4 | p=0.798  Favours null |
|  |  | 1. de Morton (4)  2. 2007  3. Australia | 1. General medicine  2. Control n=126  Intervention n=110 | 1. 78 (7)  2. 54  3. Unknown | 1. 80 (8)  2. 55.5  3. Unknown | Usual care | 1. TUG (s)  2. Within 48 hours of admission  3. At discharge | 1. 30 (28)  2. 26 (21) | 3. -5 (10) | 1. Physiotherapy-designed exercises  2. Individualised progressive exercise training – lower limb, upper limb, and trunk. | 1. 35 (30)  2. 36 (65) | 3. -10 (19) | +0.5 | p=0.63  Favours null |
|  |  | 1. Opasich (5)  2. 2010  3. Italy | 1. Cardiac surgery  2. Control n=80  Intervention n=160 | 1. 75.0 (3.9)  2. 45  3. Unknown | 1. 74.6 (3.6)  2. 40  3. Unknown | Traditional physiotherapy (including bicycle, treadmill options) | 1. a. TUG (s),  b. 6MWT (m)  2. Within 2 days of admission  3. At discharge | 1. a. 14.3 (6.2)  b. 195.2 (86)  2. a. 11.1  b. 309.2 | 3. a. -3.2 (4)  b. +114.0 (88)  4. a p<0.001  b. p<0.001 | 1. Individualised physical training programme  2. Exercises stratified by frailty ranging from assisted walking to treadmill and bicycle use. | 1. a. 13.6 (6.1)  b. 218.0 (92)  2. a. 8.8  b. 336.5 | 3. a. -4.8 (5.3)  b. +118.5 (80)  4. a p<0.001  b. p<0.001 | a. +0.4  b.  -0.1 | a. p<0.001  **Favours experimental**  b. p=0.65  Favours null |
|  |  | 1. Schwenk (6)  2. 2014  3. Germany | 1. Geriatric rehabilitation  2. Control n=74  Intervention n=74 | 1. 83.9 (6.1)  2. 76.2  3. Unknown | 1. 84.2 (6.2)  2. 83.6  3. Unknown | Usual care | 1. Gait speed (m/s)  2. 1-2 days after admission  3. 1-2 days before discharge | 1. 0.73 (0.39)  2. 0.89 (0.37) | 3. +34.9% (53.8)  4. p<0.001 | 1. Individualised physical training programme  2. Progressively increased machine-based resistance, balance, functional exercises. | 1. 0.73 (0.39)  2. 0.93 (0.38) | 3. +43.3% (53.7)  4. p<0.001 | +0.2 | p=0.354  Favours null |
|  |  | 1. Giangregorio (7)  2. 2009  3. Canada | 1. Orthopaedic rehabilitation  2. Control n=7 Intervention n=14 | 1. 83.7 (8.6)  2. 85.7  3. Unknown | 1. 79.9 (7.0)  2. 85.7  3. Unknown | Usual care | 1. TUG (s)  2. Following recruitment  3. At discharge | 1. 45.6 (10.3)  2. 20.7 (5.0) | 3. -24.9 (6.9) | 1. Body weight supported treadmill training  2. Treadmill and suspension system with progressively increased duration. | 1. 73.5 (29.1)  2. 25.2 (29.1) | 3. -48.3 | +4.4 | p=0.32  Favours null |
|  |  | 1. Zinglersen (8)  2. 2018  3. Denmark | 1. Geriatric medicine  2. Control n=48  Intervention n=20 | 1. 83.3 (8.1)  2. 75  3. 25.8 (5.3) | 1. 84.9  2. 75  3. 25.6 (4.7) | Historical control group (usual care) | 1. Gait speed (m/s)  2. Within 2 days of admission  3. Day of discharge or maximum 10 days | 1. 0.60 (0.20)  2. 0.65 | 3. +0.05  4. p=0.36 | 1. Chair based functional training  2. Individualised training – progressively increased repetitions of chair stands, reduced height of blocks (with or without NMES). | 1. 0.50 (0.20)  2. 0.61 | 3. +0.11  4. p<0.01 | NA | Not statistically different  Favours null |
|  |  | 1. Moseley (9)  2. 2009  3. Australia | 1. Orthopaedic rehabilitation  2. Control n=80  Intervention n=80 | 1. 84 (8)  2. 81.3  3. 23.4 | 1. 84 (8)  2. 81.3  3. 24.0 | Low dose (limited) weight-bearing exercise | 1. Gait speed (m/s)  2. Recruitment  3. Four weeks follow-up | 1. 0.28 (0.16)  2. 0.48 (0.22) | 3. +0.20 (0.14) | 1. Weight-bearing exercise  2. Progressive reduced support (harness whilst inpatient), increased repetition | 1. 0.30 (0.22)  2. 0.53 (0.25) | 3. +0.23 | +0.2 | p=0.345  Favours null |
|  |  | 1. Busch (10)  2. 2012  3. Germany | 1. Cardiac surgery  2. Control n=64 Intervention n=57 | 1. 78.6 (3.2)  2. 71  3. 26.8 | 1. 78.5 (3.2)  2. 67  3. 27.9 | Usual care (thrice weekly walks, calisthenics, ergometer) | 1. a. TUG (s)  b. 6MWT (m)  2. Before rehabilitation  3. At discharge | 1. a. 10 (3)  b. 311 (80)  2. a. 9 (4)  b. 352 (82) | 3. a. -1 (3)  b. +42 (52)  4. a. p<0.001 | 1. Resistance and balance training  2. Weight machines for lower limb exercises, free weights for upper limb; balls and platforms for balance | 1. a. 11 (3)  b. 296 (84)  2. a. 8 (2)  b. 363 (86) | 3. a. -2 (2)  b. +67 (49)  4. a. p<0001 | a. +0.3  b. +0.5 | a. p=0.003  **Favours experimental**  b. p=0.008  **Favours experimental** |
|  |  | 1. Raymond (11)  2. 2017  3. Australia | 1. Geriatric medicine  2. Control n=232  Intervention n=236 | 1. 84.1 (6.9)  2. 57.8  3. Unknown | 1. 84.5  2. 63.1  3. Unknown | Usual care (physiotherapy 5 days/ week) | 1. TUG(s)  2. Prior to randomisation  3. Within 48 hours prior to discharge | 1. 29 (95% CI 23-42)  2. 24 (95% CI 17-34) | 3. -5 | 1. High intensity group exercise  2. Group classes thrice weekly focussing on progressive resistance, balance exercise | 1. 29 (95% CI 20-42)  2. 22 (95% CI 16-33) | 3. -7 | NA | p=0.47  Favours null |
|  |  | 1. McCullagh (12)  2. 2017  3. Ireland | 1. General medicine  2. Control n=95  Intervention n=95 | 1. 81.7 (7.3)  2. 41  3. 26.8 (6.8) | 1. 79.7 (7.5)  2. 64  3. 26.3 (6.5) | Sham exercise programme (gentle stretching and relaxation) | 1. a. SPPB  b. Gait speed (m/s)^b^  2. Within 48 hours of admission  3. Within 24 hours of discharge | 1. a. 3 (95% CI 2 to 4)  b. 0.32 (0.18)  2. a. 3.0 (2.1)  b. 0.30 (0.20) | 3. a. +0  b. -0.02 (0.20) | 1. Augmented prescribed exercise programme  2. Individually progressed lower limb and core strengthening exercises. | 1. a. 3 (95% CI 2 to 5)  b. 0.30 (0.18)  2. a. 4.6 (2.5)  b. 0.25 (0.14) | 3. a. +1.6  b. -0.05 | a. NA  b.  -0.2 | a. p=0.003  **Favours experimental**  b. Favours null |
|  |  | 1. Martinez-Velilla (13)  2. 2019  3. Spain | 1. Geriatric medicine  2. Control n=185  Intervention n=185 | 1. 87.1 (5.2)  2. 58.9  3. 26.9 (4.9) | 1. 87.6 (4.6)  2. 54.1  3. 27.1 (4.4) | Usual care | 1. a. Gait speed (m/s)^b^  b. SPPB  2. Start of intervention  3. At discharge | 1. a.0.46 (0.20)  b. 4.7 (2.7)  2. a. 0.48 (0.19)  b. 4.9 | 3. a.+0.01 (0.12)  b. +0.2 (95% CI -0.1 to 0.5) | 1. Multicomponent physical exercise  2. Individualised progressive resistance (machines, weights), balance, walking exercises. | 1. a.0.48 (0.19)  b. 4.4 (2.5)  2. a. 0.61 (0.22)  b. 6.8 | 3. a.+0.12 (0.13)  b. +2.4 (95% CI 2.1 to 2.7) | a. +0.9  b. NA | a. p<0.001  **Favours experimental**  b. p<0.001  **Favours experimental** |
|  |  | 1. Jones (14)  2. 2006  3. Australia | 1. General medicine  2. Control n=80  Intervention n=80 | 1. 82.9 (7.6)  2. 61.3  3. Unknown | 1. 81.9 (8.0)  2. 53.8  3. Unknown | Usual care | 1. TUG (s)  2. Within 48 hours of admission  3. Within 24 hours of discharge | 1. 21.5 (95% CI 16.9 to 25.9)  2. 20.3 | 3. -1.2 (95% CI -0.9 to 4.3)  4. p=0.012 | 1. Individualised progressive exercise  2. Strengthening and mobility exercise ranging from bed to stairs exercise. | 1. 24.2 (95% CI 15.8 to 37.3)  2. 18.8 | 3. -5.4 (95% CI -1.0 to -12.4)  4. p=0.012 | NA | p=0.081  Favours null |
|  |  | 1. Fiore^b (15)^  2. 2017  3. Canada | 1. Elective colorectal surgery  2. Control n=22  Intervention=25 | 1. 73.1 (5.9)  2. 54.5  3. Unknown | 1.72.8 (6.5)  2. 40  3. Unknown | Usual care (enhanced recovery after surgery) | 1. 6MWT (m)  2. Pre-operative  3. Four weeks post-operative | 1. 487.0 (71.6)  2. 439.5 (103.3) | 3. -47.6 (88.0)  4. p=0.034 | 1. Early mobilisation  2. Physiotherapy/ physiotherapy-trained facilitated mobilisation – thrice day review | 1. 409.0 (117.6)  2. 362.4 (174.8) | 3. -46.6 (116.2)  4. p=0.067 | +0.0 | p=0.977  Favours null |
|  |  | 1. Tal-Akabi^b^ (16)  2. 2007  3. Switzerland | 1. Orthopaedic rehabilitation  2. Control n=29  Intervention n=33 | 1. 74.6 (7.8)  2. 75.9  3. 25.4 (6.3) | 1. 73.7 (6.0)  2. 66.7  3. 26.8 (5.3) | Regular intensity exercise | 1.TUG (s)  2. Within 48 hours of admission  3. Within 24 hours before discharge | 1. 29.6  2. 17.6 | 3.-8  4. p<0.001 | 1. High intensity exercise  2. Individually progressive leg press functional exercise. | 1. 27.6  2. 15.9 | 3.-11.7  4. p<0.001 | NA | Not statistically different  Favours null |
|  |  | 1. Houborg (17)  2. 2006  3. Denmark | 1. Elective colorectal surgery  2. Control n=59  Intervention n=60 | 1. 72 (7)  2. 49.2  3. 26 (3) | 1. 72 (7)  2. 50  3. 26 (5) | Sham treatment – turning, repositioning, relaxation, massages | 1. Gait speed (m/s)  2. Pre-operative  3. 7 days post-operative | 1. 1.48 (0.31)  2. 1.13 | 3. -0.35 (95% CI -0.28 to -0.47)  4. p=0.98 | 1. Strength training programme  2. Progressive strength training of upper and lower limbs, mobilisation, aerobic training. | 1. 1.39 (0.38)  2. 1.04 | 3. -0.35 (95% CI -0.25 to -0.5)  4. p=0.98 | NA | Not statistically different  Favours null |
|  |  | 1. Said ^b^ (18)  2. 2018  3. Australia | 1. Geriatric rehabilitation  2. Control n=93  Intervention n=98 | 1. 81 (95% CI 77 to 87)  2. 55  3. 25.0 (95% CI 22.5 to 28.9) | 1. 81 (95% CI 77 to 88)  2. 60  3. 24.3 (95% CI 20.9 to 29.2) | Usual care and additional social activities not impacting on mobility | 1. a. Gait speed (m/s)  b. TUG (s)  2. Within 48 hours of admission  3. Within 48 hours of discharge | 1. a. 0.29 (0.26)  b. 33.8 (19.4)  2. a. 0.56 (0.28)  b. 26.2 (20.5) | 3. a.+0.27 (0.18)  b. -7.6 (14.7) | 1. Multimodal exercise programme  2. Progressive functional, balance, strength, mobility and aerobic training. | 1. a. +0.31 (0.28)  b. 31.5 (18.5)  2. a. 0.51 (0.29)  b. 24.2 (12.5) | 3. a. +0.20  b. -7.3 | a.  -0.4  b. +0.0 | a. p=0.096  Favours null  b. p=0.724  Favours null |
|  |  | 1. Sano (19)  2. 2018  3. Spain | 1. Elective orthopaedic  2. Control n=41  Intervention n=40 | 1. 75 (5.8)  2. 78.9  3. 26.6 (3.2) | 1. 75 (6.4)  2. 81.1  3. 25.9 (3.5) | Usual care (physical therapy 5 days/week for 3 weeks) | 1. a. Gait speed (m/s)  b. TUG (s)  2. Pre-operative  3. Three weeks post-operative | 1. a. 1.02 (0.21)  b. 10.81 (2.71)  2. a. 0.92 (0.22)  b. 12.22 (3.15) | 3. a. -0.10 (0.13)  b. +1.41 (1.85) | 1. Seated side tapping training  2. Five repetitions tapping outstretched arms 10 times. | 1. a. 1.02 (0.21)  b. 10.94 (2.59)  2. a. 1.03 (0.19)  b. 10.44 (1.87) | 3. a. +0.01  b. -0.50 | a. +0.8  b. +1.0 | a. p=0.001  **Favours experimental**  b. p=0.001  **Favours experimental** |
|  |  | 1. Said ^b^ (20)  2. 2012  3. Australia | 1. Geriatric rehabilitation  2. Control n=24  Intervention n=22 | 1. 81.6 (6.5)  2. 40  3. Unknown | 1. 80.8 (4.6)  2. 59  3. Unknown | Usual care | 1. TUG (s)  2. Within 48 hours of admission  3. Within 48 hours of discharge | 1. 31.3 (12.4)  2. 32.6 (17.4) | 3. +1.3 (10.3) | 1. Enhanced physical activity  2. Increasing mobility activities evenings and weekends. | 1. 35.5 (11.8)  2. 36.8 (26.7) | 3. +1.3 | 0 | Not statistically different  Favours null |
|  |  | 1. Prasciene (21)  2. 2019  3. Lithuania | 1. Cardiac surgery  2. Control n=15  Intervention n=14 | 1. 76.5 (2.1)  2. 60  3. Unknown | 1. 72.8 (2.0)  2. 40  3. Unknown | Standard care – comprehensive three week exercise-based rehabilitation | 1. a. SPPB  b. 6MWT  2. Before rehabilitation  3. After rehabilitation | 1. a. 7.5 (0.6)  b. 242.3 (32.1)  2. a. 8.4 (0.7)  b. 341.8 (30.3) | 3. a. +0.9  b. +99.5 (23.0) | 1. Additional exercise  2. Additional exercise session three days/week including balance and resistance training | 1. a. 8.4 (0.6)  b. 239.3 (28.6)  2. a. 9.6 (0.7)  b. 359.0 (27.1) | 3. a. +1.2  b. +119.7 (20.5) | a. NA  b. +0.9 | a. p=0.202  Favours null  b. p=0.677  Favours null |
|  |  | 1. Ortiz-Alonso (22)  2. 2019  3. Spain | 1. Geriatric medicine  2. Control n=131  Intervention n=150 | 1. 88 (5)  2. 54  3. 26.0 (6.4) | 1. 88 (5)  2. 60  3. 26.1 (9.3) | Usual care | 1. SPPB  2. Admission  3. Discharge | 1. 3.8 (2.9)  2. 4.1 | 3. +0.3 (2.2) | 1. Exercise programme  2. Chair rises and walking | 1. 3.2 (2.5)  2. 3.6 | 3. +0.4 (1.8) | +0.0 | p=0.796  Favours null |
|  |  | 1. Deer (23)  2. 2019  3. USA | 1. General Medicine  2. Control n=20  3. Intervention n=21 | 1. 75.7 (7.1)  2. 70  3. 29.0 (5.3) | 1. 77.6 (7.5)  2. 67  3. 27.4 (6.4) | Placebo isocaloric supplement, usual care | 1. SPPB  2. During hospitalisation  3. Four weeks follow-up | 1. 7.8 (3.7)  2. 9.1 | 3. +1.3 (1.9) | 1. Rehabilitation  2. Chair-based exercises and resistance exercise | 1. 7.1 (2.9)  2. 9.1 | 3. +2.0 (1.9) | +0.4 | p=0.03  *(all interventions vs. placebo)*  **Favours experimental** |
|  |  | 1. Braun (24)  2. 2019  3. Germany | 1. Geriatric medicine  2.Control n=18  Intervention n=17 | 1. 83.1 (7.4)  2. 72  3. Unknown | 1. 78.6  2. 76  3. Unknown | Usual care | 1. a. Gait speed (m/s)  b. TUG (s)  c. 6MWT (m)  2. After randomisation  3. 14 days after admission | 1. a. 0.60 (0.19)  b. 24.9 (11.1)  c. 167.7 (79.4)  2. a. 0.64 (0.28)  b. 22.4 (9.5)  c. 170.8 (79.9) | 3. a. +0.04 (0.15)  b. -2.5 (5.9)  c. +3.1 (37.7) | 1. Augmented Prescribed Exercise Program  2. Individually tailored exercises from chair-based to endurance and/or walking | 1. a. 0.53 (0.17)  b. 28.6 (13.2)  c. 154.5 (59.6)  2. a. 0.65 (0.20)  b. 22.8 (12.2)  c. 194.9 (85.8) | 3. a. +0.12 (0.20)  b. -5.8 (6.6)  c. +40.4 (80.9) | a. +0.5  b. +0.6  c. +1.0 | a. p=0.25  Favours null  b. p=0.21  Favours null  c. p=0.11  Favours null |
|  | Muscle strength | 1. Sherrington (2)  2. 2003  3. Australia | 1. Orthopaedic rehabilitation  2. Control n=39  Intervention n=41 | 1. 81.1 (8.3)  2. 69  3. Unknown | 1. 81.0 (7.0)  2. 66  3. Unknown | Non-weight-bearing exercise | 1. Knee extension (kg)  2. After randomisation  3. Two weeks after baseline | 1. 94.9 (44.1)  2. 109.1 (50.8) | 3. +14.2 (32.8) | 1. Weight-bearing exercise  2. Individually progressed stepping exercises – increased repetitions, lessening hand support, increasing height of blocks. | 1. 112.0 (63.1)  2. 118.7 (61.7) | 3. +6.7 | -0.2 | p=0.14  Favours null |
|  |  | 1. Rahmann (3)  2. 2009  3. Australia | 1. Elective orthopaedic  2. Control n=20  Intervention n=24 | 1. 70.4 (9.2)  2. 70.6  3 28.8 (6.2) | 1. 69.4 (6.5)  2. 44.4  3. 28.4 (4.6) | Ward-based physiotherapy | 1. Knee extension (kg)  2. Preoperative  3. Two weeks postoperative | 1. 10.1 (4.8)  2. 8.6 (4.6) | 3. -1.5 (3.2) | 1. Aquatic physiotherapy  2. Progressive trunk stability, backstroke kick, arm swing exercises in water | 1. 14.8 (6.2)  2. 12.2 (4.2) | 3. -2.6 | -0.3 | p=0.030  Favours control |
|  |  | 1. Rahmann (3)  2. 2009  3. Australia | 1. Elective orthopaedic  2. Control n=24  Intervention n=21 | 1. 69.4 (6.5)  2. 44.4  3. 28.4 (4.6) | 1. 69.0 (8.9)  2. 43.2  3. 28.0 (4.1) | Aquatic physiotherapy | 1. Knee extension (kg)  2. Pre-operative  3. Two weeks postoperative | 1. 14.8 (6.2)  2. 12.2 (4.2) | 3. -2.6 (4.0) | 1. Water exercise  2. Progressive trunk stability, backstroke kick, arm swing exercises in water | 1. 11.5 (6.0)  2. 9.7 (3.7) | 3. -1.8 | +0.2 | p=0.456  Favours null |
|  |  | 1. Schwenk (6)  2. 2014  3. Germany | 1. Geriatric rehabilitation  2. Control n=74 Intervention n=74 | 1. 83.9 (6.1)  2. 76.2  3. Unknown | 1. 84.2 (6.2)  2. 83.6  3. Unknown | Usual care | 1. Handgrip (kg)  2. 1-2 days after admission  3. 1-2 days before discharge | 1. 14.6 (6.2)  2. 15.1 (6.6) | 3. +5.7% (21.0)  4. p=0.084 | 1. Individualised physical training programme  2. Progressively increased machine-based resistance, balance, functional exercises | 1. 14.6 (6.2)  2. 14.8 (6.7) | 3. +6.6% (43.9)  4. p=0.084 | +0.0 | p=0.834  Favours null |
|  |  | 1. Torres-Sánchez (25)  2. 2017  3. Spain | 1. Respiratory  2. Control n=29  Intervention n=29 | 1. 72.2 (8.2)  2. 31.0  3. 29.1 (2.5) | 1.76.7 (6.3)  2. 24.1  3. 31.3 (1.8) | Usual care | 1. Knee extension (kg)  2. Second day of admission  3. Day of discharge | 1. 10.3 (1.4)  2. 8.8 (4.1) | 3. -1.5 (3.8) | 1. Pedal exercises  2. Individualised progressive time, velocity, and resistance of pedal exercises. | 1. 10.6 (11.2)  2. 11.6 (3.8) | 3. +1.0 | +0.7 | p=0.028  **Favours experimental** |
|  |  | 1. Moseley (9)  2. 2009  3. Australia | 1. Orthopaedic rehabilitation  2. Control n=80  Intervention n=80 | 1. 84 (8)  2. 81.3  3. 23.4 | 1. 84 (8)  2. 81.3  3. 24.0 | Low dose (limited) weight-bearing exercise | 1. Knee extension (kg)  2. Recruitment  3. Four weeks follow-up | 1. 6.8 (3.4)  2. 7.7 (4.0) | 3. +0.9 (2.6) | 1. Weight-bearing exercise  2. Progressive reduced support (harness whilst inpatient), increased repetition | 1. 7.4 (3.3)  2. 7.8 (3.9) | 3. +0.4 | -0.2 | p=0.853  Favours null |
|  |  | 1. Busch (10)  2. 2012  3. Germany | 1. Cardiac surgery  2. Control n=64 Intervention n=57 | 1. 78.6 (3.2)  2. 71  3. 26.8 | 1. 78.5 (3.2)  2. 67  3. 27.9 | Usual care (thrice weekly walks, calisthenics, ergometer) | 1. Knee extension (kg)  2. Before rehabilitation  3. At discharge | 1. 30.2 (12.0)  2. 34.7 (14.8) | 3. +4.5 (9.5)  4. p<0.001 | 1. Resistance and balance training  2. Weight machines for lower limb exercises, free weights for upper limb; balls and platforms for balance | 1. 33.8 (13.3)  2. 39.6 (16.0) | 3. +5.8  4. p<0.001 | +0.1 | p=0.49  Favours null |
|  |  | 1. Martinez-Velilla (13)  2. 2019  3. Spain | 1. Geriatric medicine  2. Control n=185  Intervention n=185 | 1. 87.1 (5.2)  2. 58.9  3. 26.9 (4.9) | 1. 87.6 (4.6)  2. 54.1  3. 27.1 (4.4) | Usual care | 1. Handgrip (kg)  2. Start of intervention  3. At discharge | 1. 17.0 (8.0)  2. 16.2 | 3. -0.8 (95% CI -1.2 to -0.5; SD 2.4) | 1. Multi-component physical exercise  2. Individualised progressive resistance (machines, weights), balance, walking exercises. | 1. 17 (6)  2. 18.5 | 3. +1.5 (95% CI 1.1 to 1.8) | +1.0 | p<0.001  **Favours experimental** |
|  |  | 1. Blanc-Bisson^b^ (26)  2.2008  3.France | 1. Geriatric medicine  2. Control n=24  Intervention n=22 | 1. 83.9 (6.6)  2. 78.9  3. 22.9 (4.7) | 1. 86.6 (5.0)  2. 65.8  3. 25.1 (5.4) | Usual care | 1. Handgrip strength (kg)  2. At recruitment  3.‘Clinical stability’ – mean 12.4 (4.7) days | 1. 15.8 (8.6)  2. 16.8 (7.6) | 3.+0.6 (4.7) | 1. Early physiotherapy  2. Progressive exercise in bed then upright when able to stand. | 1. 16.1 (6.9)  2. 16.8 (8.0) | 3. +1.0 (3.4) | +0.1 | p=0.753  Favours null |
|  |  | 1. Henriksen^b^ (27)  2. 2002  3. Denmark | 1. Elective colorectal surgery  2. Control n=12  Intervention n=13 | 1. 74.4 (4.0)  2. 33.3  3. Unknown | 1. 73.5 (5.5)  2. 69.2  3. Unknown | Normal mobilisation by nursing staff | 1. a. Handgrip strength (kg)  b. Knee extension (kg)  2. Pre-operative  3. 7 days post-operative | 1. a. 38.1 (12.9)  b. 35.2 (10.4)  2. a. 38.1 (12.9)  b. 33.9 (10.1) | 3. a. -2.8 (3.4)  b. -5.2 (3.9) | 1. Enhanced recovery  2. Early mobilisation | 1. a. 28.9 (10.2)  b. 22.9 (9.9)  2. a. 28.3 (10.0)  b. 22.7 (8.3) | 3. a. -0.7 (1.1)  b. -0.2 (4.0) | a. +0.6  b. +1.3 | a. p=0.100  Favours null  b. p=0.042  **Favours experimental** |
|  |  | 1. Houborg (17)  2. 2006  3. Denmark | 1. Elective colorectal surgery  2. Control n=59  Intervention n=60 | 1. 72 (7)  2. 49.2  3. 26 (3) | 1. 72 (7)  2. 50  3. 26 (5) | Sham treatment – turning, repositioning, relaxation, massages | 1. a. Knee extension (kg)  b. Handgrip (kg)  2. Pre-operative  3. 7 days post-operative | 1. a. 30.0 (12.5)  b. 30.4 (13.0)  2. a. 23.4  b. 28.4 | 3. a. -6.6 (95% CI -4.1 to 9.2)  b. -2.0 (95% CI 0 to -3.6)  4. a. p=0.14  b. p=0.42 | 1. Strength training programme  2. Progressive strength training of upper and lower limbs, mobilisation, aerobic training. | 1. a.28.2 (12.0)  b. 29.1 (12.2)  2. a. 24.1  b. | 3. a. -4.1 (95% CI -1.5 to -6.6)  b. -3.1 (95% CI -1.0 to 5.1)  4. a. p=0.14  b. p=0.42 | NA | a. Not statistically different  Favours null  b. Not statistically different  Favours null |
|  |  | 1.McGowan ^b^ (28)  2. 2018  3. UK | 1. Acute medicine for older people  2. Control n=25 Intervention n=25 | 1.82.9 (5.7)  2. 54.2  3. Unknown | 1. 87.1 (9.2)  2. 66.7  3. Unknown | Usual care | 1. a. Knee extension (kg)  b. Knee flexion (kg)  2. Within 48 hours of admission  3. At 7 days or discharge if earlier. | 1. a. 8.9 (2.7)  b. 9.6 (2.9)  2. a. 9.0 (3.5)  b. 9.2 (2.7) | 3. a. -0.4 (2.7)  b. +0.1 (3.2) 4. a. p=0.887  b. p=0.321 | 1. Pedal exerciser  2. Thrice daily pedal exercises, five minutes each. | 1. a. 8.1 (2.4)  b. 9.8 (2.9) | 3. a. +0.3 (2.2)  b. +0.2 (2.1)  4. a. p=0.588  b. p=0.714 | a. +0.3  b. +0.0 | a. p=0.851  Favours null  b. p=309  Favours null |
|  |  | 1. McCullagh (12)  2. 2017  3. Ireland | 1. General medicine  2. Control n=95  Intervention n=95 | 1. 81.7 (7.3)  2. 41  3. 26.8 (6.8) | 1. 79.7 (7.5)  2. 64  3. 26.3 (6.5) | Sham exercise programme (gentle stretching and relaxation) | 1. Handgrip (kg)  2. Within 48 hours of admission  3. Within 24 hours of discharge | 1. 17.0 (7.8)  2. 18.1 (7.0) | 3. +1.1 (2.9) | 1. Augmented prescribed exercise programme  2. Individually progressed lower limb and core strengthening exercises. | 1. 16.9 (7.6)  2. 18.1 (7.4) | 3. +1.2 | +0.0 | Favours null |
|  |  | 1. Sano (19)  2. 2018  3. Spain | 1. Elective orthopaedic  2. Control n=41  Intervention n=40 | 1. 75 (5.8)  2. 78.9  3. 26.6 (3.2) | 1. 75 (6.4)  2. 81.1  3. 25.9 (3.5) | Usual care (physical therapy 5 days/week for 3 weeks) | 1. a. Knee extension (kg)  b. Knee flexion (kg)  2. Pre-operative  3. Three weeks post-operative | 1. a. 16.0 (6.7)  b. 9.2 (4.5)  2. a. 7.8 (3.3)  b. 6.9 (2.4) | 3. a. -8.2 (4.7)  b. -2.3 (3.7) | 1. Seated side tapping training  2. Five repetitions tapping outstretched arms 10 times. | 1. a. 16.2 (7.1)  b. 9.5 (3.8)  2. a. 7.7 (2.9)  b. 6.7 (2.4) | 3. a. -8.5  b. -2.8 | a.  -0.1  b.  -0.1 | a. p=0.883  Favours null  b. p=0.654  Favours null |
|  | Muscle mass | 1. Deer (23)  2. 2019  3. USA | 1. General Medicine  2. Control n=20  3. Intervention n=21 | 1. 75.7 (7.1)  2. 70  3. 29.0 (5.3) | 1. 77.6 (7.5)  2. 67  3. 27.4 (6.4) | Placebo isocaloric supplement, usual care | 1. DXA FFM (kg)  2. During hospitalisation  3. Four weeks follow-up | 1. 44.6 (9.8)  2. 45.1 | 3. +0.5 (0.9) | 1. Rehabilitation  2. Chair-based exercises and resistance exercise | 1. 42.7 (9.8)  2. 43.6 | 3. +0.9 (1.8) | +0.4 | p=0.72  *(all interventions vs. placebo)*  Favours null |
| Nutrition | Physical performance | 1. Niccoli (29)  2. 2017  3. Canada | 1. Geriatric medicine  2. Control n=26  Intervention n=26 | 1. 80.3(1.6)  2. 68  3. 26.4 (6.6) | 1.81.8 (1.7)  2. 68.2  3. 24.2 (5.2) | Usual care | 1. a. TUG (s)  b. Gait speed (m/s)  2. At recruitment  3. Prior to discharge | 1. a. 0.56 (0.06)  b. 28.2 (2.8)  2. a. 0.74 (0.06)  b. 21.8 (2.2) | 3. a. +0.18 (0.03)  b. -6.4 (1.9)  4. a. p<0.001  b. p=0.002 | 1. Whey protein  2. Whey protein mixed into cereal and/or milk products. | 1. a. 0.52 (0.03)  b. 28.3 (3.1)  2. a. 0.66 (0.04)  b. 21.2 (2.0) | 3. a. +0.15 (0.04)  b. -7.1 (2.2)  4. a. p<0.001  b. p=0.003 | a. +1.0  b. +0.4 | Not statistically different  Favours null |
|  |  | 1. Beelen (30)  2. 2017  3. Netherlands | 1. General medicine  2. Control n=39  Intervention n=36 | 1. 77.2 (7.2)  2. 56.4  3. 28.2 (5.6) | 1. 76.5 (6.7)  2. 55.6  3. 26.9 (6.1) | Regular non-enriched variants of intervention products | 1. SPPB  2. Within 2 days of hospital admission  3. Two weeks post-discharge | 1. 6.0 (2.5)  2. 6.9 (2.5) | 3. +0.9 | 1. Protein-enriched familiar foods  2. Patients could order food choices from menu without knowledge of enrichment. | 1. 6.8 (0.4)  2. 7.2 (0.5) | 3. +0.4 | NA | Not statistically different  Favours null |
|  |  | 1. Gade (31)  2. 2019  3. Denmark | 1. General medicine  2. Control n=82  Intervention n=83 | 1. 84.2 (6.3)  2. 65.3  3. 25.8 (5.2) | 1. 85.3 (6.2)  2. 69.9  3. 25.1 (4.2) | Placebo isoenergetic beverage, resistance exercise, vitamin D | 1. Gait speed (m/s)  2. Within 72 hours of admission  3. Within 72 hours of discharge | 1. 0.5 (IQR 0.4 -0.7)  2. 0.5 | 3. +0.0 (IQR -0.1 – 0.7) | 1. Protein-enriched milk-based supplement  2. After breakfast and resistance exercise, vitamin D | 1. 0.6 (IQR 0.5 – 0.9)  2. 0.6 | 3. +0.0 (IQR -0.1 – 0.1) | NA | p=0.481  Favours null |
|  |  | 1. Pedersen (32)  2. 2019  3. Denmark | 1. General medicine  2. Control n=42 Intervention n=43 | 1. 82.5 (7.5)  2. 60.5  3. 24.5 (95% CI 22.3 – 30.0) | 1. 82.1 (7.4)  2. 71.4  3. 25.3 (95% CI 22.3 – 29.1) | Standard care | 1. Gait speed (m/s)  2. On admission  3. Within first week after discharge | 1. 0.6 (95% CI 0.5 – 0.8)  2. 0.7 (95% CI 0.5 – 0.8) | 3. +0.1 | 1. Protein and exercise  2. Progressive strength training and immediate protein supplementation | 1. 0.6 (95% CI 0.4 – 0.8)  2. 0.7 (95% CI 0.5 – 0.9) | 3. +0.1 | NA | p=0.06  Favours null |
|  |  | 1. Deer (23)  2. 2019  3. USA | 1. General Medicine  2. Control n=20  3. Intervention n=20 | 1. 75.7 (7.1)  2. 70  3. 29.0 (5.3) | 1. 80.0 (8.7)  2. 70  3. 28.9 (6.4) | Placebo isocaloric supplement, usual care | 1. SPPB  2. During hospitalisation  3. Four weeks follow-up | 1. 7.8 (3.7)  2. 9.1 | 3. +1.3 (1.9) | 1. Whey protein  2. Twice daily 20g whey protein | 1. 6.2 (3.1)  2. 8.9 | 3. +2.7 (2.3) | +0.7 | p=0.03  *(all interventions vs. placebo)*  **Favours experimental** |
|  |  | 1. Deer (23)  2. 2019  3. USA | 1. General Medicine  2. Control n=20  3. Intervention n=20 | 1. 75.7 (7.1)  2. 70  3. 29.0 (5.3) | 1. 80.0 (8.8)  2. 70  3. 26.1 (6.6) | Placebo isocaloric supplement, usual care | 1. SPPB  2. During hospitalisation  3. Four weeks follow-up | 1. 7.8 (3.7)  2. 9.1 | 3. +1.3 (1.9) | 1. Whey protein and rehabilitation  2. Chair-based exercises and resistance exercise and whey protein | 1. 6.2 (3.5)  2. 9.6 | 3. +3.4 (2.1) | +1.1 | p=0.03  *(all interventions vs. placebo)*  **Favours experimental** |
|  |  | 1. Files (33)  2. 2020  3. USA | 1. Critical care  2. Control n=11  Intervention n=11 | 1. 67.9 (2.6)  2. 45.5  3. 31.3 (1.7) | 1. 69.1 (3.2)  2. 45.5  3. 30.6 (1.9) | Placebo nitrate-depleted beetroot juice | 1. SPPB  2. ICU discharge  3. Hospital discharge or 14 days after visit 1 | 1. 0 (IQR 0 – 2)  2. 3 (IQR 2 – 6) | 3. +3 | 1. Nitrate-rich beetroot juice  2. Once/ day for 14 days | 1. 0 (IQR 0 – 0.5)  2. 0 (IQR 0 – 5.5) | 3. +0.0 | NA | p=0.14)  Favours null |
|  | Muscle strength | 1. Beelen (30)  2. 2017  3. Netherlands | 1. General medicine  2. Control n=39  Intervention n=36 | 1. 77.2 (7.2)  2. 56.4  3. 28.2 (5.6) | 1. 76.5 (6.7)  2. 55.6  3. 26.9 (6.1) | Regular non-enriched variants of intervention products | 1. a. Handgrip (kg)  b. Knee extension (kg)  2. Within 2 days of hospital admission  3. Two weeks post-discharge | 1. a. 25.5 (12.5)  b. 22.2 (4.4)  2. a. 24.6 (8.1)  b. 22.7 (4.8) | 3. a. -0.9 (5.8)  b. +0.5 (3.1) | 1. Protein-enriched familiar foods  2. Patients could order food choices from menu without knowledge of enrichment. | 1. a. 27.1 (9.6)  b. 23.1 (4.3)  2. a. 25.3 (8.4)  b. 24.5 (4.7) | 3. a. -1.8  b. +2.4 | a.  -0.2  b. +0.3 | Not statistically different  Favours null |
|  |  | 1. Ekinci (34)  2. 2016  3. Turkey | 1. Orthopaedic surgery  2. Control n=37  Intervention n=38 | 1. 83.1 (7.1)  2. 100  3. 22.3 (2.7) | 1. 82.2 (7.3)  2. 100  3. 21.8 (2.1) | Standard post-operative nutrition | 1. Handgrip (kg)  2. Pre-operative  3. 15 days post-operative | 1. 5.3 (3.4)  2. 6.3 (3.7) | 3. +1.0 (0.4)  4. p=0.001 | 1. Beta-hydroxy-beta-methylbutyrate (HMB) supplementation  2. Two servings HMB enriched products in addition to standard nutrition | 1. 7.1 (4.0)  2. 7.8 (4.1) | 3. +0.7  4. p=0.001 | -0.8 | p=0.338  Favours null |
|  |  | 1. Hermanky (35)  2. 2017  3. Austria | 1. Orthopaedic surgery  2. Control n=20  Intervention n=20 | 1. 79.9 (8.5)  2. 65  3. 24.8 (4.1) | 1. 79.1 (9.3)  2. 66.7  3. 26.3 (5.9) | Usual care (limited details) | 1.Handgrip (kg)  2. Within 48 hours of admission  3. At discharge | 1. 22.5 (8.4)  2. 21.6 (7.9) | 3. -0.9 (3.1)  4. p=0.041 | 1. Nutrition and exercise  2. Nutritional consultation to reach defined energy and protein intake with moderate strength training. | 1. 18.6 (6.2)  2. 18.9 (6.0) | 3. -0.3  4. p=0.166 | +0.2 | p=0.570  Favours null |
|  |  | 1. Saudny-Unterberger (36)  2. 1997  3. Canada | 1. Respiratory  2. Control n=16  Intervention n=17 | 1. 69.4 (3.9)  2. 30  3. 25.7 | 1. 69.2 (2.2)  2. 43  3. 23.4 | Food ordered from hospital menu | 1. Handgrip (kg)  2. At admission  3. Two weeks post admission | 1. 26.0 (2.8)  2. 26.4 | 3. +0.4 (0.9) | 1. Oral nutritional supplements  2. Nutritional supplements and snacks between meals. | 1. 29.7 (3.0)  2. 28.8 | 3. -0.9 (1.0) | -1.4 | p=0.385  Favours null |
|  |  | 1. Niccoli (29)  2. 2017  3. Canada | 1. Geriatric medicine  2. Control n=26  Intervention n=26 | 1. 80.3(1.6)  2. 68  3. 26.4 (6.6) | 1.81.8 (1.7)  2. 68.2  3. 24.2 (5.2) | Usual care | 1. a. Handgrip (kg)  b. Knee extension (kg)  2. At recruitment  3. Prior to discharge | 1. a. 15.3 (1.4)  b. 15.8 (1.0)  2. a. 15.8 (1.4)  b. 17.3 (1.1) | 3. a. +0.7 (0.5)  b. +1.5 (0.4)  4. a. 0.244  b. p=0.170 | 1. Whey protein  2. Whey protein mixed into cereal and/or milk products. | 1. a. 13.4 (1.2)  b. 12.3 (1.6)  2. a. 15.0 (1.1)  b. 15.2 (1.3) | 3. a. +2.0 (0.8)  b. +2.9 (1.8)  4. a. p<0.001  b. p=0.032 | a. +2.6  b. +3.5 | a. p=0.455  Favours null  b. p=0.071  Favours null |
|  |  | 1. Gade (31)  2. 2019  3. Denmark | 1. General medicine  2. Control n=82  Intervention n=83 | 1. 84.2 (6.3)  2. 65.3  3. 25.8 (5.2) | 1. 85.3 (6.2)  2. 69.9  3. 25.1 (4.2) | Placebo isoenergetic beverage, resistance exercise, vitamin D | 1. Handgrip (kg)  2. Within 72 hours of admission  3. Within 72 hours of discharge | 1. 17.7 (IQR 13.1 – 22.7)  2. 17.4 | 1. -0.3 (IQR -2.2 - +2.3) | 1. Protein-enriched milk-based supplement  2. After breakfast and exercise | 1. 17.8 (IQR 13.3 – 23.3)  2. 18.0 | 3. +0.2 (IQR-1.9 - +0.6) | NA | p=0.681  Favours null |
|  |  | 1. Pedersen (32)  2. 2019  3. Denmark | 1. General medicine  2. Control n=42 Intervention n=43 | 1. 82.5 (7.5)  2. 60.5  3. 24.5 (95% CI 22.3 – 30.0) | 1. 82.1 (7.4)  2. 71.4  3. 25.3 (95% CI 22.3 – 29.1) | Standard care | 1. Handgrip (kg)  2. On admission  3. Within first week after discharge | 1. 21.1 (8.7)  2. 21.8 (8.9) | 3. +0.7 (3.3) | 1. Protein and exercise  2. Progressive strength training and immediate protein supplementation | 1. 21.5 (10.3)  2. 23.5 (9.9) | 3. +2.0 (3.8) | +0.4 | p=0.008  **Favours experimental** |
|  | Muscle mass | 1. Hermanky (35)  2. 2017  3. Austria | 1. Orthopaedic surgery  2. Control n=20  Intervention n=20 | 1. 79.9 (8.5)  2. 65  3. 24.8 (4.1) | 1. 79.1 (9.3)  2. 66.7  3. 26.3 (5.9) | Usual care (limited details) | 1.Bioelectrical Impedance Analysis – Fat Free Mass (kg)  2. Within 48 hours of admission  3. At discharge | 1. Unknown  2. Unknown | 3. -1.347  4. p=0.162 | 1. Nutrition and exercise  2. Nutritional consultation to reach defined energy and protein intake with moderate strength training. | 1. Unknown  2. Unknown | 3. -0.324  4. p=0.626 | NA | Favours null |
|  |  | 1. Ogasawara (37)  2. 2018  3. Japan | 1. Respiratory medicine  2. Control n=21  Intervention n=21 | 1. 79.1 (6.8)  2. 4.8  3. 19.1 (2.8) | 1. 79.5 (8.1)  2. 14.3  3. 19.3 (2.4) | Similar energy oral nutritional supplements free of EPA | 1. Bioelectrical Impedance Analysis – Skeletal Muscle Index (kg/m^2^)  2. Admission  3. Discharge | 1. 5.9 (1.0)  2. 5.6 (14) | 3. +0.3  4. p=0.35 | 1. EPA-enriched oral nutritional supplements  2. One can or pack given each day | 1. 6.0 (1.1)  2. 6.2 (1.1) | 3.+ 0.2  4. p=0.13 | NA | p=0.10  Favours null |
|  |  | 1. Bouillanne (38)  2. 2018  3. France | 1. Geriatric medicine rehabilitation  2. Control n=14 Intervention n=13 | 1. 88 (95% CI 77-92)  2. 76.9  3. 21.6 (95% CI 18.2-33.2) | 1. 89 (95% CI 74-97)  2. 72.7  3. 19.7 (95% CI 16.4-26.5) | Placebo – mixture of six non-essential amino acids | 1. DEXA – Appendicular Skeletal Muscle Mass (kg)  2. At recruitment  3. Day 20 | 1. 14.1 (95% CI 10.9-21.6)  2. 14.0 (95% CI 11.6-24.9) | 3. -0.1 | 1. Citrulline amino acid  2. 10g citrulline given once a day for 21 days | 1. 11.8 (95% CI 9.9-20.4)  2. 13.3 (95% CI 11.4-18.5) | 3. +1.5 | NA | p=0.83  Favours null |
|  |  | 1. Deer (23)  2. 2019  3. USA | 1. General Medicine  2. Control n=20  3. Intervention n=20 | 1. 75.7 (7.1)  2. 70  3. 29.0 (5.3) | 1. 80.0 (8.7)  2. 70  3. 28.9 (6.4) | Placebo isocaloric supplement, usual care | 1. DXA FFM (kg)  2. During hospitalisation  3. Four weeks follow-up | 1. 44.6 (9.8)  2. 45.1 | 3. +0.5 (0.9) | 1. Whey protein  2. Twice daily 20g whey protein | 1. 42.1 (8.9)  2. 42.2 | 3. +0.1 (1.6) | -0.4 | p=0.72  *(all interventions vs. placebo)*  Favours null |
|  |  | 1. Deer (23)  2. 2019  3. USA | 1. General Medicine  2. Control n=20  3. Intervention n=20 | 1. 75.7 (7.1)  2. 70  3. 29.0 (5.3) | 1. 80.0 (8.8)  2. 70  3. 26.1 (6.6) | Placebo isocaloric supplement, usual care | 1. DXA FFM (kg)  2. During hospitalisation  3. Four weeks follow-up | 1. 44.6 (9.8)  2. 45.1 | 3. +0.5 (0.9) | 1. Whey protein and rehabilitation  2. Chair-based exercises and resistance exercise and whey protein | 1. 40.3 (9.7)  2. 40.9 | 3. +0.6 (1.6) | +0.1 | p=0.72  *(all interventions vs. placebo)*  Favours null |
|  |  | 1. Gade (31)  2. 2019  3. Denmark | 1. General medicine  2. Control n=82  Intervention n=83 | 1. 84.2 (6.3)  2. 65.3  3. 25.8 (5.2) | 1. 85.3 (6.2)  2. 69.9  3. 25.1 (4.2) | Placebo isoenergetic beverage, resistance exercise, vitamin D | 1. Bioelectrical Impedance Analysis – Lean Body Mass  2. Within 72 hours of admission  3. Within 72 hours of discharge | 1. 42.5 (IQR 38.6 – 52.2)  2. 41.5 | 3. -0.1 (IQR -1.3 - +0.8) | 1. Protein-enriched milk-based supplement  2. After breakfast and exercise | 1. 44.0 (IQR 36.6 – 49.9)  2. 44.7 | 3. -0.3 (IQR -2.1 - +0.8) | NA | p=0.332  Favours null |
| Pharmaceutical | Physical Performance | 1. Deer (23)  2. 2019  3. USA | 1. General Medicine  2. Control n=20  3. Intervention n=19 | 1. 75.7 (7.1)  2. 70  3. 29.0 (5.3) | 1. 77.1 (7.4)  2. 74  3. 27.1 (5.3) | Placebo isocaloric supplement, usual care | 1. SPPB  2. During hospitalisation  3. Four weeks follow-up | 1. 7.8 (3.7)  2. 9.1 | 3. +1.3 (1.9) | 1. Testosterone  2. Single IM dose | 1. 7.4 (3.2)  2. 10.2 | 3. +2.8 (2.1) | +0.8 | p=0.03  *(all interventions vs. placebo)*  **Favours experimental** |
|  | Muscle strength | 1. Weissberger (39)  2. 2003  3. UK | 1. Elective orthopaedic  2. Control n=16  Intervention n=17 | 1. 67.3 (1.5)  2. 68.8  3. 27.1 (1.1) | 1. 70.1 (1.6)  2. 70.6  3. 26.2 (1.2) | Placebo injection | 1. Knee flexion  2. Pre-operatively at start of treatment  3. Four weeks post-operatively | 1. Unknown  2. Unknown | 3. -4.2% | 1. Growth hormone  2. Once daily injections for 14 weeks preoperatively | 1. Unknown  2. Unknown | 3. -1.7% | NA | p=0.004  **Favours experimental** |
|  |  | 1. Hedström (40)  2. 2004  3. Sweden | 1. Orthopaedic surgery  2. Control n=9 Intervention n=11 | 1. 85 (3)  2. 75  3. 20.4 (1.8) | 1. 83 (7)  2. 75  3. 22.8 (4.5) | Placebo | 1.Knee extension (Nm)  2. Before treatment  3. End of treatment (21-28 days) | 1. 10.5 (5.1)  2. 12.5 | 3. +2.0 (3.4) | 1. Recombinant human growth hormone  2. Once daily subcutaneous injection for 21-28 days | 1. 10.5 (4.1)  2. 12.9 | 3. +2.4 (3.1) | +0.1 | p=0.8  Favours null |
|  | Muscle mass | 1. Hedström (40)  2. 2004  3. Sweden | 1. Orthopaedic surgery  2. Control n=9 Intervention n=11 | 1. 85 (3)  2. 75  3. 20.4 (1.8) | 1. 83 (7)  2. 75  3. 22.8 (4.5) | Placebo | 1. DEXA – Lean Body Mass (kg)  2. Before treatment  3. End of treatment (21-28 days) | 1. 38.1 (5.6)  2. 34.9 | 3. -3.2 | 1. Recombinant human growth hormone  2. Once daily subcutaneous injection for 21-28 days | 1. 39.9 (6.2)  2. 39.2 | 3. -0.6 | NA | p=0.03  **Favours experimental** |
|  |  | 1. Weissberger (39)  2. 2003  3. UK | 1. Elective orthopaedic  2. Control n=16  Intervention n=17 | 1. 67.3 (1.5)  2. 68.8  3. 27.1 (1.1) | 1. 70.1 (1.6)  2. 70.6  3. 26.2 (1.2) | Placebo injection | 1. Thigh cross sectional area  2. Pre-operatively at start of treatment  3. Four weeks post-operatively | 1. Unknown  2. Unknown | 3. -10.1% | 1. Growth hormone  2. Once daily injections for 14 weeks preoperatively | 1. Unknown  2. Unknown | 3. +2.3% | NA | p=0.43  Favours null |
|  |  | 1. Deer (23)  2. 2019  3. USA | 1. General Medicine  2. Control n=20  3. Intervention n=19 | 1. 75.7 (7.1)  2. 70  3. 29.0 (5.3) | 1. 77.1 (7.4)  2. 74  3. 27.1 (5.3) | Placebo isocaloric supplement, usual care | 1. DXA FFM (kg)  2. During hospitalisation  3. Four weeks follow-up | 1. 44.6 (9.8)  2. 45.1 | 3. +0.5 (0.9) | 1. Testosterone  2. Single IM dose | 1. 39.9 (6.4)  2. 39.6 | 3. -0.3 (1.4) | -0.9 | p=0.72  *(all interventions vs. placebo)*  Favours null |
|  |  | 1. Zhang (41)  2. 2019  3. China | 1. Orthopaedics  2. Control n=33  Intervention n=44 | 1. 78.6 (7.7)  2. 100  3. Unknown | 1. 79.5 (6.2)  2. 100  3. Unknown | Usual care | 1. DXA ASM (kg)  2. Before surgery  3. Four weeks post-operatively | 1. 12.4 (1.2)  2. 12.5 (1.3) | 3. +0.1 | 1. Erythropoietin injections  2. IM injections once daily for 10 days from day of surgery | 1. 12.8 (1.5)  2. 13.0 (1.7) | 3. +0.2 | NA | p<0.001  **Favours experimental** |
|  |  | 1. Zhang (41)  2. 2019  3. China | 1. Orthopaedics  2. Control n=25  Intervention n=39 | 1. 75.0 (8.2)  2. 0  3. Unknown | 1. 77.0 (7.7)  2. 0  3. Unknown | Usual care | 1. DXA ASM (kg)  2. Before surgery  3. Four weeks post-operatively | 1. 18.4 (1.8)  2. 18.4 (1.9) | 3. +0.0 | 1. Erythropoietin injections  2. IM injections once daily for 10 days from day of surgery | 1. 18.6 (1.7)  2. 18.8 (1.8) | 3. +0.2 | NA | p<0.001  **Favours experimental** |
|  |  | 1. Sloan (42)  2. 1992  3. Canada | 1. Orthopaedic surgery  2. Control n=14  Intervention n=15 | 1. 81 (6)  2. 100  3. Unknown | 1. 83 (7)  2. 100  3. Unsknown | Placebo injections | 1. Bioelectrical Impedance Analysis – Lean Body Weight (kg)  2. Within 48 hours of surgery  3. At four weeks or discharge | 1. 35.6 (4.7)  2. 31.5 (4.8) | 3. -4.1 | 1. Nandrolone decanoate injection  2. IM injection 2mg/kg weekly for four weeks | 1. 31.6 (4.4)  2. 30.6 (3.2) | 3. -1.0 | NA | Not significantly different  Favours null |
| Neuromuscular Electrical Stimulation (NMES) | Physical performance | 1. Zinglersen (8)  2. 2018  3. Denmark | 1. Geriatric medicine  2. Control n=8  Intervention n=12 | 1. 84.9  2. 75  3. 26.1 (5.5) | 1. 81.8 (8.9)  2. 75  3. 25.2 (4.1) | Functional training alone | 1. Gait speed (m/s)  2. Within 2 days of admission  3. Day of discharge or maximum 10 days | 1. 0.50 (0.20)  2. 0.61 | 3. +0.11 | 1. NMES  2. NMES combined with functional training (as detailed in physical activity section) | 1. 0.50 (0.20)  2. 0.60 | 3. +0.10 | NA | Not significant  Favours null |
|  |  | 1. Lopez-Lopez (43)  2. 2019  3. Spain | 1. General medicine  2. Control n=47  Intervention n=48 | 1. 72.5  2. 42.6  3. 30.5 (5.7) | 1. 74.9  2. 58.3  3. 25.9 (4.4) | Standard care | 1. SPPB  2. Day of admission  3. At discharge | 1. 4.2 (4.1)  2. 4.2 (3.2) | 3. -0.0 (1.7)  4. p=0.563 | 1. NMES with rehabilitation  2. NMES with increasing levels of exercise | 1. 3.6 (4.2)  2. 5.9 (3.6) | 3. +2.3 (2.4)  4. p<0.001 | +1.4 | p=0.027  **Favours experimental** |
|  | Muscle strength | 1. Martin-Salvador (44)  2. 2016  3. Spain | 1. Respiratory  2. Control n=20 Intervention n=24 | 1. 77.4 (5.2)  2. 22  3. 28.9 (5.2) | 1. 78.8 (6.3)  2. 16.8  3. 27.6 (3.8) | Usual care | 1. Knee extension (kg)  2. Admission  3. Discharge | 1. 10.5 (5.0)  2. 9.0 (3.4) | 3. -1.5 (3.2)  4. p=0.005 | 1. Exercise and NMES combined  2. 30 minutes daily electric stimulation of both quadriceps | 1. 11.1 (3.1)  2. 11.8 (4.2) | 3. -0.7  4. p=0.408 | +0.3 | p=0.008  **Favours experimental** |

1. Wnuk BR, Durmala J, Ziaja K, Kotyla P, Wozniewski M, Blaszczak E. A Controlled Trial of the Efficacy of a Training Walking Program in Patients Recovering from Abdominal Aortic Aneurysm Surgery. Advances in clinical and experimental medicine : official organ Wroclaw Medical University. 2016;25(6):1241-371.

2. Sherrington C, Lord SR, Herbert RD. A randomised trial of weight-bearing versus non-weight-bearing exercise for improving physical ability in inpatients after hip fracture. Australian Journal of Physiotherapy. 2003;49(1):15-22.

3. Rahmann AE, Brauer SG, Nitz JC. A specific inpatient aquatic physiotherapy program improves strength after total hip or knee replacement surgery: a randomized controlled trial. Archives of Physical Medicine & Rehabilitation. 2009;90(5):745-55.

4. de Morton NA, Keating JL, Berlowitz DJ, Jackson B, Lim WK. Additional exercise does not change hospital or patient outcomes in older medical patients: a controlled clinical trial. Australian Journal of Physiotherapy. 2007;53(2):105-11.

5. Opasich C, Patrignani A, Mazza A, Gualco A, Cobelli F, Pinna GD. An elderly-centered, personalized, physiotherapy program early after cardiac surgery. European journal of cardiovascular prevention and rehabilitation : official journal of the European Society of Cardiology, Working Groups on Epidemiology & Prevention and Cardiac Rehabilitation and Exercise Physiology. 2010;17(5):582-7.

6. Schwenk M, Dutzi I, Englert S, Micol W, Najafi B, Mohler J, et al. An intensive exercise program improves motor performances in patients with dementia: translational model of geriatric rehabilitation. Journal of Alzheimer's disease : JAD. 2014;39(3):487-98.

7. Giangregorio LM, Thabane L, DeBeer J, Farrauto L, McCartney N, Adachi JD, et al. Body weight-supported treadmill training for patients with hip fracture: a feasibility study. Archives of Physical Medicine & Rehabilitation. 2009;90(12):2125-30.

8. Zinglersen AH, Halsteen MB, Kjaer M, Karlsen A. Can electrical stimulation enhance effects of a functional training program in hospitalized geriatric patients? Experimental gerontology. 2018;106:101‐8.

9. Moseley AM, Sherrington C, Lord SR, Barraclough E, St George RJ, Cameron ID. Mobility training after hip fracture: a randomised controlled trial. Age Ageing. 2009;38(1):74-80.

10. Busch JC, Lillou D, Wittig G, Bartsch P, Willemsen D, Oldridge N, et al. Resistance and balance training improves functional capacity in very old participants attending cardiac rehabilitation after coronary bypass surgery. Journal of the american geriatrics society. 2012;60(12):2270‐6.

11. Raymond MJ, Jeffs KJ, Winter A, Soh SE, Hunter P, Holland AE. The effects of a high-intensity functional exercise group on clinical outcomes in hospitalised older adults: an assessor-blinded, randomised-controlled trial. Age and ageing. 2017;46(2):208‐13.

12. McCullagh R. A randomised controlled trial to measure the effects of an augmented prescribed exercise programme (APEP) on length of stay, physical ability and quality of life in frail older medical patients in the acute setting: University of Cork; 2017.

13. Martínez-Velilla NC-H, Alvaro Zambom-Ferraresi, Fabricio Sáez de Asteasu, Mikel L. Lucia, Alejandro Galbete, Arkaitz García-Baztán, Agurne Alonso-Renedo, Javier González-Glaría, Belen Gonzalo-Lázaro, María Apezteguía Iráizoz, Itziar Gutiérrez-Valencia, Marta Rodríguez-Mañas, Leocadio Izquierdo, Mikel López Sáez de Asteasu, Mikel. Effect of Exercise Intervention on Functional Decline in Very Elderly Patients During Acute Hospitalization: A Randomized Clinical Trial. JAMA Internal Medicine. 2019;179(1):28-36.

14. Jones CT, Lowe AJ, MacGregor L, Brand CA, Tweddle N, Russell DM. A randomised controlled trial of an exercise intervention to reduce functional decline and health service utilisation in the hospitalised elderly. Australasian Journal on Ageing. 2006;25(3):126-33.

15. Fiore JF, Jr., Castelino T, Pecorelli N, Niculiseanu P, Balvardi S, Hershorn O, et al. Ensuring Early Mobilization Within an Enhanced Recovery Program for Colorectal Surgery: A Randomized Controlled Trial. Ann Surg. 2017;266(2):223-31.

16. Tal-Akabi A, Steiger U, Villiger PM. Neuromuscular adaptation to early post-operative, high-intensity, short resistance training of non-operated lower extremity in elderly patients: a randomized controlled trial. Journal of Rehabilitation Medicine (Stiftelsen Rehabiliteringsinformation). 2007;39(9):724-9.

17. Houborg KB, Jensen MB, Hessov I, Laurberg S. Little effect of physical training on body composition and nutritional intake following colorectal surgery--a randomised placebo-controlled trial. Eur J Clin Nutr. 2005;59(8):969-77.

18. Said CM, Morris ME, McGinley JL, Szoeke C, Workman B, Liew D, et al. Additional structured physical activity does not improve walking in older people (> 60 years) undergoing inpatient rehabilitation: a randomised trial. Journal of Physiotherapy (Elsevier). 2018;64(4):237-44.

19. Sano YI, A. Wanaka, H. Matsui, M. Yamamoto, S. Koyanagi, J. Iwata, H. An easy and safe training method for trunk function improves mobility in total knee arthroplasty patients: a quasi-randomized controlled trial. Plos one. 2018;13(10) (no pagination).

20. Said CM, Morris ME, Woodward M, Churilov L, Bernhardt J. Enhancing physical activity in older adults receiving hospital based rehabilitation: a phase II feasibility study. BMC geriatrics. 2012;12:26.

21. Prasciene E, Icking N, Kisieliute E, Kubilius R, Bjarnason-Wehrens B. Feasibility and short-term effectiveness of an additional resistance and balance training in cardiac rehabilitation for older patients after valve surgery: results of a pilot study. EuroPrevent 2019; Portugal: European Journal of Preventive Cardiology; 2019. p. S39.

22. Ortiz-Alonso J, Bustamante-Ara N, Valenzuela PL, Vidán-Astiz M, Rodríguez-Romo G, Mayordomo-Cava J, et al. Effect of a Simple Exercise Program on Hospitalization-Associated Disability in Older Patients: A Randomized Controlled Trial. Journal of the American Medical Directors Association. 2020;21(4):531-7.e1.

23. Deer RR, Dickinson JM, Baillargeon J, Fisher SR, Raji M, Volpi E. A Phase I Randomized Clinical Trial of Evidence-Based, Pragmatic Interventions to Improve Functional Recovery After Hospitalization in Geriatric Patients. The Journals of Gerontology: Series A. 2019;74(10):1628-36.

24. Braun T, Grüneberg C, Süßmilch K, Wiessmeier M, Schwenk I, Eggert S, et al. An augmented prescribed exercise program (APEP) to improve mobility of older acute medical patients – a randomized, controlled pilot and feasibility trial. BMC Geriatrics. 2019;19(1):240.

25. Torres-Sanchez I, Valenza MC, Cabrera-Martos I, Lopez-Torres I, Benitez-Feliponi A, Conde-Valero A. Effects of an Exercise Intervention in Frail Older Patients with Chronic Obstructive Pulmonary Disease Hospitalized due to an Exacerbation: a Randomized Controlled Trial. COPD: journal of chronic obstructive pulmonary disease. 2017;14(1):37‐42.

26. Blanc-Bisson C, Dechamps A, Gouspillou G, Dehail P, Bourdel-Marchasson I. A randomized controlled trial on early physiotherapy intervention versus usual care in acute care unit for elderly: potential benefits in light of dietary intakes. Journal of nutrition, health & aging. 2008;12(6):395‐9.

27. Henriksen MG, Jensen MB, Hansen HV, Jespersen TW, Hessov I. Enforced mobilization, early oral feeding, and balanced analgesia improve convalescence after colorectal surgery. Nutrition. 2002;18(2):147-52.

28. McGowan T, Ong T, Kumar A, Lunt E, Sahota O. The effect of chair-based pedal exercises for older people admitted to an acute hospital compared to standard care: a feasibility study. Age & Ageing. 2018;47(3):483-6.

29. Niccoli S, Kolobov A, Bon T, Rafilovich S, Munro H, Tanner K, et al. Whey Protein Supplementation Improves Rehabilitation Outcomes in Hospitalized Geriatric Patients: a Double Blinded, Randomized Controlled Trial. Journal of nutrition in gerontology and geriatrics. 2017;36(4):149‐65.

30. Beelen J, de Roos NM, de Groot LCPGM. A 12-week intervention with protein-enriched foods and drinks improved protein intake but not physical performance of older patients during the first 6 months after hospital release: a randomised controlled trial. British Journal of Nutrition. 2017;117(11):1541-9.

31. Gade J, Beck AM, Andersen HE, Christensen B, Rønholt F, Klausen TW, et al. Protein supplementation combined with low-intensity resistance training in geriatric medical patients during and after hospitalisation: a randomised, double-blind, multicentre trial. The British journal of nutrition. 2019;122(9):1006-20.

32. Pedersen MM, Petersen J, Beyer N, Larsen HG, Jensen PS, Andersen O, et al. A randomized controlled trial of the effect of supervised progressive cross-continuum strength training and protein supplementation in older medical patients: the STAND-Cph trial. Trials. 2019;20(1):655.

33. Files DC, Heinrich T, Shields KL, Love NJ, Brailer C, Bakhru RN, et al. A randomized pilot study of nitrate supplementation with beetroot juice in acute respiratory failure. Nitric Oxide. 2020;94:63-8.

34. Ekinci O, Yanık S, Terzioğlu Bebitoğlu B, Yılmaz Akyüz E, Dokuyucu A, Erdem Ş. Effect of Calcium β-Hydroxy-β-Methylbutyrate (CaHMB), Vitamin D, and Protein Supplementation on Postoperative Immobilization in Malnourished Older Adult Patients With Hip Fracture. Nutrition in Clinical Practice. 2016;31(6):829-35.

35. Hermanky M, Korninger C, Fuchs D, Strasser B. Effects of a protein-optimized diet in combination with moderate strength training on the postoperative course in elderly patients with hip fracture. Aktuel Ernahrungsmed. 2017;42(03):180-7.

36. Saudny-Unterberger H, Martin JG, Gray-Donald K. Impact of nutritional support on functional status during an acute exacerbation of chronic obstructive pulmonary disease. Am J Respir Crit Care Med. 1997;156(3 Pt 1):794-9.

37. Ogasawara T, Marui S, Miura E, Sugiura M, Matsuyama W, Aoshima Y, et al. Effect of eicosapentaenoic acid on prevention of lean body mass depletion in patients with exacerbation of chronic obstructive pulmonary disease: A prospective randomized controlled trial. Clinical nutrition ESPEN. 2018;28:67-73.

38. Bouillanne O, Melchior JC, Faure C, Paul M, Canoui-Poitrine F, Boirie Y, et al. Impact of 3-week citrulline supplementation on postprandial protein metabolism in malnourished older patients: the Ciproage randomized controlled trial. Clinical nutrition (Edinburgh, Scotland). 2018;(no pagination).

39. Weissberger AJ, Anastasiadis AD, Sturgess I, Martin FC, Smith MA, Sönksen PH. Recombinant human growth hormone treatment in elderly patients undergoing elective total hip replacement. Clinical endocrinology. 2003;58(1):99‐107.

40. Hedström M, Sääf M, Brosjö E, Hurtig C, Sjöberg K, Wesslau A, et al. Positive effects of short-term growth hormone treatment on lean body mass and BMC after a hip fracture: a double-blind placebo-controlled pilot study in 20 patients. Acta orthopaedica scandinavica. 2004;75(4):394‐401.

41. Zhang Y, Chen L, Wu P, Lang J, Chen L. Intervention with erythropoietin in sarcopenic patients with femoral intertrochanteric fracture and its potential effects on postoperative rehabilitation. Geriatrics & Gerontology International. 2020;20(2):150-5.

42. Sloan JP, Wing P, Dian L, Meneilly GS. A pilot study of anabolic steroids in elderly patients with hip fractures. Journal of the american geriatrics society. 1992;40(11):1105‐11.

43. López-López L, Torres-Sánchez I, Rodríguez-Torres J, Cabrera-Martos I, Ortiz-Rubio A, Valenza MC. Does adding an integrated physical therapy and neuromuscular electrical stimulation therapy to standard rehabilitation improve functional outcome in elderly patients with pneumonia? A randomised controlled trial. Clin Rehabil. 2019;33(11):1757-66.

44. Martin-Salvador A, Colodro-Amores G, Torres-Sanchez I, Moreno-Ramirez MP, Cabrera-Martos I, Valenza MC. Physical therapy intervention during hospitalization in patients with acute exacerbation of chronic obstructive pulmonary disease and pneumonia: A randomized clinical trial. Medicina clinica. 2016;146(7):301-4.

| **Author, date** | **Inclusion criteria** | **Exclusion criteria** |
| --- | --- | --- |
| **Physical activity** | | |
| Busch (10), 2012 | • ≥75 years  • Coronary artery disease  • Complete revascularisation after bypass graft  • Able to start cardiac rehabilitation within 4 weeks after surgery  • 6MWT 100 – 350m | • Exercise limiting comorbidities (e.g. orthopaedic or neurological)  • Heart failure NYHA Class IV  • Haemoglobin <90g/L  • Wound healing disturbance  • Cognitive or linguistic deficits  • Peripheral artery occlusive disease |
| Blanc-Bisson^b^ (26)^,^ 2008 | • >70years  • Confined to bed or transferring from bed to chair with assistance  • Independent locomotion within 3 months | • Neuromuscular diseases affecting lower limbs  • Chronic respiratory failure  • Heart failure NYHA Class IV  • Peripheral vascular disease  • Palliative care  • Use of muscle-impairing drugs |
| Braun (24), 2019 | • ≥65 years  • Planned acute geriatrics stay ≥2weeks  • Able to walk independently (with/without aid) or standby assistance  • TUG > 9 sec | • Significant cognitive impairment  • Severe hearing or visual impairment  • Language barrier  • Acute psychiatric problem  • Palliative care  • Any medical restriction on interventions |
| de Morton (4), 2007 | • ≥65 years  • Admitted to either of two medical wards with a general medical condition | • Admitted from nursing home  • Assessed to need nursing home level or palliative care  • Stroke or condition for which mobilisations contraindicated (e.g. fracture)  • Too unwell to ambulate or exercise  • Readmitted following previous participation in study |
| Deer (23), 2019 | • ≥65 years  • Residing at home before/ after admission  • Self-reported ability to walk across small room two weeks before admission  • Able to stand independently at baseline testing | • Uncontrolled hypertension  • History of stroke with motor disability  • Renal or liver insufficiency  • Anabolic steroids within 3 months  • Planned hospitalisation within 30 days of discharge  • Cognitive impairment  • Living more than 30miles from hospital |
| Fiore^b^ (15), 2017 | • >18years  • Planned colorectal resection | • Known metastases  • Neurological or musculoskeletal conditions that preclude postoperative mobilisation  • Unable to speak English or French  • Critical care admission straight after surgery |
| Giangregorio (7), 2009 | • Treated surgically for hip fracture  • Stable fracture or adequate fixation  • Able to follow two step commands  • Able to take few steps with help of assistive device | • In isolation  • Cultures positive for MRSA  • Able to walk without assistive devices  • Hip, knee, or ankle surgery before hip fracture  • Unable to give informed consent  • Incontinent  • Uncontrolled cardiovascular disease, Diabetes Mellitus, or hypertension  • Neuromuscular disease or other musculoskeletal disease |
| Henriksen^b^ (27)^,^ 2002 | • Referred for elective colorectal surgery | • Inflammatory bowel disease  • Disseminated cancer  • Serious cardiopulmonary disease |
| Houborg (17), 2006 | • ≥60 years  • Referred for elective colorectal surgery | • Living more than 40km from hospital  • Inflammatory bowel disease  • Disseminated cancer  • Significant psychiatric disease or dementia  • Other medical reasons that precluded physical training |
| Jones (14), 2006 | • ≥65 years  • General medical admission  • Able to give informed consent | • Nursing home resident or nursing level of care at home  • Medically unstable  • Mobilisation contraindicated by treating medical team  • Admitted to delirium management unit  • Non weight-bearing  • Requiring palliative care  • Diagnosis known to cause functional impairment (e.g. stroke, fracture)  • Expected LoS <24 hours |
| Martinez-Velilla (13), 2019 | • ≥75 years  • Barthel index ≥60  • Able to ambulate with/ without assistance  • Able to communicate and collaborate with research team | • Expected LoS <6 days  • Very severe cognitive decline  • Terminal illness  • Uncontrolled arrhythmias  • Acute pulmonary embolism  • Recent myocardial infarction  • Recent major surgery  • Extremity bone fracture in the past 3 months |
| McCullagh (12), 2017 | • ≥65 years  • General medical admission  • Anticipated LoS >3days  • Needed either an aid or assistance to walk on admission | • Medically too unwell  • Contra-indication to exercise present e.g. hip fracture, uncontrolled heart rate  • Assistance of more than one person to walk safely required  • Baseline SPPB 0 or 1  • Admitted for surgical, critical, end of life, or psychiatric care  • Unable to follow commands in English (language or too confused/ agitated)  • Participated in trial within previous 12 months |
| McGowan ^b^ (28), 2018 | • ≥65 years  • Admitted to hospital within preceding 48 hr  • Able to sit in a chair independently  • Able to follow 1-stage command | • Predicted discharge within next 48hr  • Terminally ill or moribund  • Needing isolation precautions  • Bedbound prior to admission  • Condition that made them unable to use pedal exerciser |
| Moseley (9), 2009 | • Surgical fixation for hip fracture admitted to rehabilitation wards  • Approval to weightbear or partial weightbear  • Able to tolerate exercise programmes  • Able to take ≥4 steps with forearm support frame and assistance of one person  • No medical contraindications limiting ability to exercise  • Living at home or low care residential facility prior to fracture with plan to return on discharge | • >4 adjusted errors on Short Portable Mental Status Questionnaire and no carer available to supervise exercise programme  • Discharged directly from acute orthopaedic ward |
| Ortiz-Alonso (22), 2019 | • >75 years-old  • Admitted to acute care of elderly unit during recruitment dates | • Nonambulatory or dependent in all ADLs 2weeks before admission  • Unstable cardiovascular disease or other major condition contraindicating exercise  • Terminal illness  • Dementia  • LoS <3days  • Death prior to discharge  • Scheduled admission  • Transferred from another hospital |
| Opasich (5), 2010 | • >70 years  • Medically stable patients (i.e. without acute diseases such  as acute heart failure, systemic infection, acute respiratory  failure etc.)  • Admitted to unit after cardiac surgery | • Mini Mental State Examination score < 20 |
| Prasciene (21), 2019 | • ≥65 years  • Valve surgery or intervention  • Ability to start rehabilitation within 4weeks of surgery  • 6MWT 100-350m  • Written consent | Not specified |
| Rahmann (3), 2009 | • Planned primary hip or knee replacement for osteoarthritis  • Home visit not possible prior to admission | • Diagnosed neurologic disorder  • Another major musculoskeletal disorder that altered mobility  • Cognitive dysfunction  • Undergoing revision joint surgery or bilateral knee replacements  • Specifically requested aquatic physiotherapy postoperatively and not willing to be randomised |
|  |  |  |
| Raymond (11), 2017 | • ≥65 years  • Admitted to geriatric medicine ward  • Able to participate in weight-bearing exercise i.e. adequate exercise tolerance, able to stand from chair with minimum/no assistance | • Medical instability  • Pre-morbidly non-ambulant  • Mini Mental State Examination score <10  • Admitted for palliation  • Weight-bearing restrictions  • Planned discharge < 7 days  • Inappropriate behaviour or cognition for group exercises |
| Said ^b^ (20), 2012 | • ≥60 years  • Improve mobility/ walking listed as goal on admission | • Primary reason for admission was to await residential care placement  • Did not require physiotherapy  • Medical restrictions on mobilisation  • Non-English speaking and advocate not available |
| Said ^b^ (18), 2018 | • Admitted to four participating geriatric rehab wards at two hospitals  • Aged > 60  • Goal to "improve mobility or walking" determined by admission referral or treating therapist  • Informed consent was obtained from the participant or ‘responsible  person’ within 48 hours of admission, with interpreters utilised  as necessary | • Medical restrictions limiting mobilisation  • Goals were non-weightbearing  • Enrolled in another randomised trial  • Primary reason for admission was carer training or residential care placement |
| Sano (19), 2018 | • >60 years  • Able to walk >10m without assistance one week after total knee arthroplasty | • Any medical or neurological problem affecting ability to complete trial e.g. stroke, cardiac insufficiency, acute respiratory failure |
| Schwenk (6), 2014 | • Dementia confirmed  • Written informed consent or informed legal guardian  • Age > 65  • No delirium  • No aphasia  • No severe visual or auditory impairment  • No severe psychiatric disorders  • No contraindications for intensive resistance and functional training such as orthopaedic instability, hernia, or uncontrolled disorders | • No additional exclusion criteria specified |
| Sherrington (2), 2003 | • Admitted to rehabilitation ward following recent fall-related hip fracture | • <60 years  • Unable to complete assessments and exercise program due to one or more of a) cognitive impairment b) major medical conditions c) complications from fracture directed to be non-weightbearing |
| Tal-Akabi^b^ (16)^,^ 2007 | • Admitted to musculoskeletal rehabilitation unit after lower limb surgery | • Neuromuscular, cardiovascular, or other disorders that could influence and/or limit participation in tailored strength training programme  • Taking corticosteroids or anabolic drugs |
| Torres-Sánchez (25), 2017 | • ≥65 years  • Admitted to respiratory ward due to acute exacerbation of Chronic Obstructive Pulmonary Disease | • Inability to provide informed consent  • Presence of psychiatric or cognitive disorders  • Severe orthopaedic problems  • Organ failure  • Cancer  • Inability to cooperate  • Another exacerbation in previous month  • Did not complete at least four days of intervention |
| Wnuk(1), 2016 | • Males aged 65-75 years  • Stable cardiovascular disease  • Absence of neurological disorders  • Non-symptomatic aneurysm | • Neurological disorders  • Unstable coronary heart disease  • Symptomatic aortic aneurysm  • Aortic dissection  • Having difficulty in locomotion  • Not able to start physical training first or second day after surgery  • Psychiatric diseases  • Lack of compliance with physiotherapist  • Other medical contraindications |
|  |  |  |
| **Nutrition** | | |
| Beelen (30), 2017 | • ≥65 years  • Admitted to general, geriatric, or respiratory medicine | • Hospital stay expected to be <4 days  • Terminally ill  • Food allergy or intolerance that restricted them from receiving standard energy and protein-rich menu/ protein-enriched intervention products  • eGFR ≤30  • Communication difficulties - aphasia or not understanding Dutch  • Delirium diagnosis  • At risk for developing refeeding syndrome |
| Bouillanne (38), 2018 | • >70 years  • Moderate undernutrition | • Severe cognitive impairment  • Severe inflammation (CRP>50)  • Diabetes mellitus  • Being fed by parenteral or enteral nutrition  • Severe renal insufficiency  • Class IV heart failure  • Severe liver disease  • Documented intestinal insufficiency  • Respiratory failure  • Chronic infectious or inflammatory disease  • Corticosteroid medications or progressive cancer |
| Deer (23), 2019 | • ≥65 years  • Residing at home before/ after admission  • Self-reported ability to walk across small room two weeks before admission  • Able to stand independently at baseline testing | • Uncontrolled hypertension  • History of stroke with motor disability  • Renal or liver insufficiency  • Anabolic steroids within 3 months  • Planned hospitalisation within 30 days of discharge  • Cognitive impairment  • Living more than 30miles from hospital |
|  |  |  |
| Ekinci (34), 2016 | • Female hip fracture patients  • ≥65 years  • Ambulatory pre-fracture  • Nutritional risk screening 2002 ≥3 and followed by hospital nutrition support team | • Diabetes Mellitus  • Organ failure  • Renal and hepatic failure  • Gastrointestinal intolerance  • Endocrine pathology e.g. thyroid disorders  • Dementia |
| Files (33), 2020 | • ≥55 years  • PaO_2_:FiO_2_ <300  • Mechanical or non-invasive ventilation  • Resolving respiratory failure | • Mechanical ventilation for > 7days  • Current hospitalisation > 14 days  • Inability to walk previously (with or without aid)  • Injury causing inability to walk or perform functional tests  • Neuromuscular disease  • Pregnancy  - Non-verbal prior to acute illness  • Acute stroke  • BMI > 50  • Body weight <= 60kg  • Cancer treatment within the last 6 months  • Moribund  • Participation in another research study  • Current use of nitroglycerine or nitrate preparations  • Current use of PDE type 5 inhibitors  • Inability to take drug by oral or  nasogastric tube  • Active gastrointestinal bleeding  • Renal replacement therapy  • Severe liver disease |
| Gade (31), 2019 | • ≥70 years  • Danish speaking  • Expected LoS >3days  • Independent stand function >30sec | • Active cancer or terminal disease  • Renal insufficiency  • Cognitive impairment  • Parenteral nutrition only  • Milk or lactose allergy or intolerance  • Weight loss plan or special diet  • Permanent Pacemaker |
| Hermanky (35), 2017 | • ≥65 years  • Surgically treated hip fracture | • Pathological fracture  • Severe renal insufficiency or the need for dialysis  • Presence of a severe cognitive impairment  • Implanted Pacemaker  • Refusal of the consumption of animal food |
| Niccoli (29), 2017 | • ≥60 years  • Ability to perform functional tests (with or without use of an assistive device)  • Willing to give informed consent and be randomised | • NYHA Class III or IV heart failure  • Clinically significant aortic stenosis, history of cardiac arrest, cardiac defibrillator, or uncontrolled angina  • Lung disease requiring oral or injected steroids or use of supplemental oxygen  • Modified mini-mental state <70  • Severe arthritis  • Cancer requiring treatment in past 3 years  • Parkinson's or other serious neurological disorders  • Renal disease requiring dialysis  • Other illness of such severity that life expectancy considered to be less than 12 months  • Current diagnosis of schizophrenia, other psychotic disorders, or bipolar disorder  • Current consumption of more than 14 alcoholic units/ week  • Clinical judgement concerning participant safety or noncompliance |
| Ogasawara (37), 2018 | • Diagnosed Chronic Obstructive Pulmonary Disease  • Hospitalised for acute exacerbation or community-acquired pneumonia  • Planned to receive pulmonary rehabilitation  • Able to eat and drink safely | • History of severe drug allergy  • Taking oral nutritional supplements during the trial  • Uncontrolled diabetes mellitus or dyslipidaemia  • Refused pulmonary rehabilitation |
| Pedersen (32), 2019 | • ≥65 years  • Admitted with acute illness from own home to emergency department | • Terminal illness or cancer treatment  • COPD  • Living outside three identified municipalities  • Unable to speak Danish  • Inability to cooperate with tests/ exercises  • Critical care admission  • Expected LoS <2days  • Inability to stand |
| Saudny-Unterberger (36), 1997 | • Consecutive patients aged 40 to 85  • Admitted to chest institute  • Diagnosis of Chronic Obstructive Pulmonary Disease  • FEV1 ≤60% predicted  • Able to give informed signed consent | • Required mechanical ventilation  • Gastrointestinal tract disorder  • Active cancer or other condition predisposing to weight loss  • Terminally ill  • Unable to communicate in English or French  • Mental confusion  • Followed a special diet |
| **Pharmaceutical** | | |
| Deer (23), 2019 | • ≥65 years  • Residing at home before/ after admission  • Self-reported ability to walk across small room two weeks before admission  • Able to stand independently at baseline testing | • Uncontrolled hypertension  • History of stroke with motor disability  • Renal or liver insufficiency  • Anabolic steroids within 3 months  • Planned hospitalisation within 30 days of discharge  • Cognitive impairment  • Living more than 30miles from hospital  • For testosterone arm: history of breast/prostate cancer, palpable prostate nodule, raised PSA, low haematocrit, decompensated heart failure |
| Hedström (40), 2004 | • >65 years  • Previously ambulant, not cognitively impaired  • Femoral neck or trochanteric fracture | • Treated with GH during the last 12 months  • Severe illness during the last 6 months  • Major surgery within 1 month  • Glaucoma  • Insulin-treated diabetes mellitus • Current or previous malignant  disease  • Severe liver or renal disease  • Known or suspected alcohol abuse  • Suspected to be non-cooperative. |
| Sloan (42), 1992 | • Elderly patients with hip fractures admitted to orthopaedic surgery | • In extended care prior to admission  • <65 years  • Severe dementia  • Severe medical illnesses e.g. congestive heart failure, metastatic cancer  • Hormone responsive tumours  • Prostatic obstruction  • Liver disease |
| Weissberger (39), 2003 | • Awaiting elective total hip replacement for osteoarthritis  • In good general health  • Without evidence of significant renal impairment, liver disease, diabetes mellitus, poorly controlled hypertension, or malignancy (past or current) | • Not meeting inclusion criteria |
| Zhang (41), 2019 | • ≥60 years  • Femoral intertrochanteric fracture  • Able to sign written consent form  • First hip surgery  • Anaemia due to surgical perioperative red blood cell mobilisation | • Diabetes Mellitus  • Ongoing cancer treatment  • Nerve or muscle dysfunction  • Other diseases caused by limited physical activity  • Serious underlying diseases |
|  |  |  |
| **Neuromuscular Electrical Stimulation** | | |
| Lopez-Lopez (43), 2019 | • Elderly patients hospitalised due to pneumonia (community-acquired) | • Hospital-acquired pneumonia  • Musculoskeletal or neurological conditions that might interfere with the evaluation or intervention  • Intervention group patients exhibiting changes in mental status  • Likely to leave hospital within 5 days  • Inability to complete any of the interventions |
| Martin-Salvador (44), 2016 | • Aged 65-90  • FVC <60%  • Admitted for community-acquired pneumonia or acute exacerbation of COPD | • Significant cognitive impairment  • Effusion, pneumothorax or haemoptysis  • Cancer  • Dermatological or venous insufficiency, with osteo-synthesis material  • Could not perform the evaluation  • In isolation  • Admitted within previous two weeks |
| Zinglersen (8), 2018 | • ≥65 years  • Admitted to geriatric medicine ward | • Cognitive impairment  • Inability to give informed consent  • Dementia  • Severe memory impairment  • Delirium  • Non-Danish speaking  • More than one assistant to enable mobilisation  • Unable to rise from chair without prominent armrest support or >9 repetitions in 30s chair stand test  • Terminal cancer, severe COPD, severe heart failure, isolated due to infectious disease  • Expected LOS < 6 days |
|  |  |  |


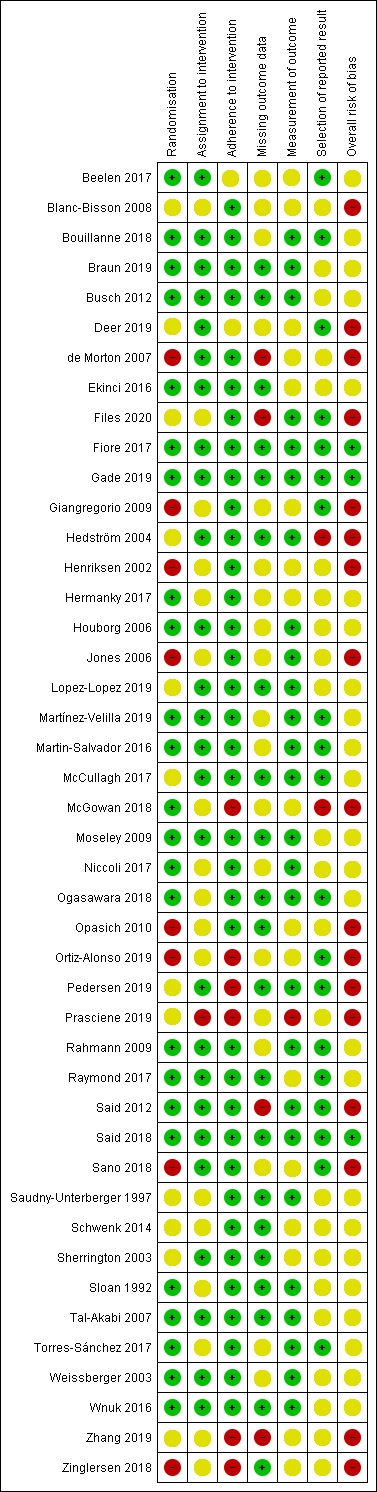


Figure S1 – Risk of bias results for each individual included study. Green circles denote low risk of bias, yellow circles denote some concerns, and red circles denote high risk of bias.
